# Position and orientation of the westerly jet determined Holocene rainfall patterns in China

**DOI:** 10.1038/s41467-019-09866-8

**Published:** 2019-05-30

**Authors:** Ulrike Herzschuh, Xianyong Cao, Thomas Laepple, Anne Dallmeyer, Richard J. Telford, Jian Ni, Fahu Chen, Zhaochen Kong, Guangxiu Liu, Kam-Biu Liu, Xingqi Liu, Martina Stebich, Lingyu Tang, Fang Tian, Yongbo Wang, Juliane Wischnewski, Qinghai Xu, Shun Yan, Zhenjing Yang, Ge Yu, Yun Zhang, Yan Zhao, Zhuo Zheng

**Affiliations:** 1 Polar Terrestrial Environmental Systems, Alfred Wegener Institute for Polar and Marine Research in the Helmholtz Association, Potsdam, 14473 Germany; 2 Institute of Environmental Sciences and Geography, University Potsdam, Potsdam, 14476 Germany; 3 Institute of Biochemistry and Biology, University Potsdam, Potsdam, 14476 Germany; 4 Max Planck Institute for Meteorology, Bundesstraße 53, 20146 Hamburg, Germany; 5 Department of Biological Sciences, University of Bergen, and Bjerknes Centre for Climate Research, Bergen, N-5020 Norway; 6 College of Chemistry and Life Sciences, Zhejiang Normal University, Jinhua, 321004 China; 7 Key Laboratory of Alpine Ecology (LAE), CAS Center for Excellence in Tibetan Plateau Earth Sciences, Institute of Tibetan Plateau Research, Chinese Academy of Science, Beijing, 100101 China; 8 Key Laboratory of Western China’s Environmental Systems (Ministry of Education), College of Earth and Environmental Sciences, Lanzhou University, Lanzhou, 730000 China; 9 State Key Laboratory of Vegetation and Environmental Change, Institute of Botany, Chinese Academy of Sciences, Beijing, 100093 China; 10 Cold and Arid Regions Environmental and Engineering Research Institute, Chinese Academy of Sciences, Lanzhou, 730000 China; 11 Department of Oceanography and Coastal Sciences, Louisiana State University, Baton Rouge, LA 70803 USA; 12 Beijing Key Laboratory of Resource Environment and GIS, College of Resource Environment and Tourism, Capital Normal University, Beijing, 100048 China; 13 Senckenberg Research Station of Quaternary Palaeontology, Weimar, 99423 Germany; 14 Nanjing Institute of Geology and Palaeontology, Chinese Academy of Sciences, Nanjing, 210008 China; 15 Arctic Research Centre & Centre for Environmental Humanities, Aarhus University, Aarhus, 8000 Denmark; 16 College of Resources and Environment Sciences, Hebei Normal University, Shijiazhuang, 050024 China; 17 Xinjiang Institute of Ecology and Geography, Chinese Academy of Sciences, Urumqi, 830011 China; 18 Institute of Hydrogeology and Environmental Geology, Chinese Academy of Geological Sciences, Shijiazhuang, China; 19 State Key Laboratory of Lake Science and Environment, Nanjing Institute of Geography and Limnology, Chinese Academy of Sciences, Nanjing, 210008 China; 20 Institute of Geographic Sciences and Natural Resources Research, Chinese Academy of Sciences, Beijing, 100101 China; 21 University of Chinese Academy of Sciences, Beijing, 100049 China; 22 School of Earth Science and Engineering, Sun Yat-sen University, Guangzhou, China; 23 Present Address: Key Laboratory of Alpine Ecology (LAE), CAS Center for Excellence in Tibetan Plateau Earth Sciences, Institute of Tibetan Plateau Research, Chinese Academy of Science, Beijing, China

**Keywords:** Climate sciences, Palaeoclimate

## Abstract

Proxy-based reconstructions and modeling of Holocene spatiotemporal precipitation patterns for China and Mongolia have hitherto yielded contradictory results indicating that the basic mechanisms behind the East Asian Summer Monsoon and its interaction with the westerly jet stream remain poorly understood. We present quantitative reconstructions of Holocene precipitation derived from 101 fossil pollen records and analyse them with the help of a minimal empirical model. We show that the westerly jet-stream axis shifted gradually southward and became less tilted since the middle Holocene. This was tracked by the summer monsoon rain band resulting in an early-Holocene precipitation maximum over most of western China, a mid-Holocene maximum in north-central and northeastern China, and a late-Holocene maximum in southeastern China. Our results suggest that a correct simulation of the orientation and position of the westerly jet stream is crucial to the reliable prediction of precipitation patterns in China and Mongolia.

## Abstract (web summary)

The basic mechanisms behind the East Asian Summer Monsoon remain poorly understood. Using proxy-based reconstructions and simulations, here the authors show that changes in the orientation and position of the westerly jet stream resulted in regionally asynchronous Holocene precipitation maxima.

## Introduction

A profound understanding of the atmospheric dynamics embedded in the East Asian Summer Monsoon (EASM), particularly of the low-level monsoonal flow that transports moist air to East Asia from nearby oceans, is of utmost importance for predicting ecological and economic changes that China is likely to face as a result of a changing climate^1^. Simulating the EASM is particularly challenging because of strong interactions between the mid-latitude and low-latitude circulation systems^2^. The monsoonal circulation is driven by numerous controls of the ocean–atmosphere system that are ultimately triggered by the annual insolation cycle^3^. The associated distinctive spatiotemporal rainfall pattern is, however, a result of interactions between the monsoon and the upper tropospheric westerly jet stream^4–7^. Ensemble simulations of future climate predict an overall warming accompanied by an increase in precipitation over northern China during the next two decades and a reduction over southeastern China^8,9^. Paleoclimate simulations using Earth System Models (ESMs) support this projection, revealing a similar precipitation pattern of change associated with increasing temperatures^6,10–12^. However, the results of these simulations conflict with proxy-based evidence of changes in Holocene precipitation, although this evidence varies between different proxies. For example, while stalagmite records throughout China (such as those from the Sanbao Cave in north-central China) consistently indicate an early-Holocene precipitation maximum^13^, high-resolution pollen-based reconstructions from north-central and northeastern China show a mid-Holocene precipitation maximum instead^14–16^ (early Holocene: 11–7 ka, mid-Holocene: 7–3 ka, late Holocene: 3–0 ka; where ka = thousands of years before present). Investigations based on sedimentary records also revealed regional differences in the timing of the precipitation maximum^17–22^ with no consistent spatiotemporal pattern among these studies. Accordingly, the proxy-based inferences were assigned to a number of different mechanisms including seasonality changes^17^, relative changes in the evaporation-to-precipitation ratio^18^, or interactions with the Indian Summer Monsoon^19^. Recently, variations in the interactions between the EASM and the westerly jet have been considered as an explanation of spatial differences in the timing of Holocene maximum^6,12,23,24^. Based on modeling results from a general circulation model it was speculated that the seasonal variations in the northward displacement of the westerly jet stream during the Holocene may have determined the length of the monsoonal period in the different regions leading to an early-Holocene maximum in northern China and a late-Holocene maximum in southern China^6,24^. However, the proxy-based reconstruction of mid-Holocene maxima in north-central and northeastern China are in conflict with these and other simulations^6,10–12^. All the proposed explanations lack a broad-scale and methodologically consistent data–model comparison approach. Accordingly, regionally varying timing of the Holocene precipitation maxima poses a major challenge to a common understanding of EASM dynamics and may have impeded an accurate representation of EASM dynamics in general circulation models. Precipitation projections in China and Mongolia for the coming decades may, therefore, involve high levels of uncertainty, despite being derived from state-of-the-art climate models.

In this paper, we present spatiotemporal precipitation patterns for the Holocene derived from pollen-based reconstructions, providing unprecedented spatial coverage for an empirical dataset that has been reconstructed using a consistent approach throughout. Similar Holocene precipitation patterns were also obtained using a minimal empirical (analog-based) model that explores, and extrapolates from, present-day relationships between insolation, westerly jet stream characteristics, and rainfall.

This study provides consistent empirical and modeling results with respect to regional variations in the timing of Holocene precipitation maxima including mid-Holocene maxima in north-central and northeastern China. This model–data agreement leads us to the conclusion that the temporal variation in the position and orientation of the westerly jet stream mainly controlled the spatiotemporal precipitation pattern—providing a new answer to the old question of circulation mechanisms behind Holocene climate change in China and Mongolia. Our simple analog-based simulation reproduces proxy-inferred Holocene precipitation trends and even quantitative precipitation anomalies as well, indicating that, irrespective of the time-scale or the forcing mechanisms invoked, spatiotemporal precipitation patterns are caused by similar mesoscale climatic processes. Our study thus implies that a better representation of these mesoscale processes and a more accurate implementation of monsoon–westerly jet interactions in ESMs will likely lead to a decrease in the uncertainty of precipitation projections.

## Results

### Pollen-based Holocene rainfall patterns in China and Mongolia

Pollen trapped in sedimentary archives provides the only quantitative proxy for continental climates on millennial-time-scales during the Holocene that has sufficient spatial resolution for detailed spatiotemporal interpretation. Modern pollen records from eastern Asia have been shown to mainly reflect annual precipitation levels^25^, because this region is largely characterized by moisture-limited vegetation (see more arguments in the Methods section). This study presents annual precipitation reconstructions for the period between 10 and 2ka, obtained by applying pollen–climate transfer functions to fossil pollen records (see Methods section). A total of 101 sets of pollen records was available from the transition between areas influenced by the monsoon and the westerly jet stream (20–55°N and 80–130°E)^26^ (Supplementary Table 1). We have assumed that changes in the Holocene pollen record provide a reliable reflection of climatic changes rather than of human impact, since the major trends in pollen-based reconstructions show a highly significant correlation with those from a semi-quantitative, non-vegetation, precipitation-proxy dataset derived from the same sites (see Supplementary Fig. 1). This assumption is also supported by results obtained in previous investigations^19,27^.

**Fig. 1:**
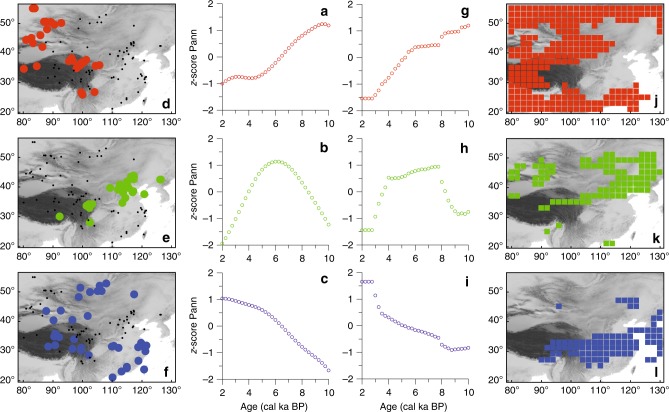
Holocene precipitation patterns for eastern Asia derived from clustering of Holocene time-series of pollen-inferred and simulated annual precipitation. **a**–**c** (pollen-infered) and **g**–**i** (simulated) show temporal variations in the centers of each cluster, as derived from unsupervised fuzzy competitive learning (see Methods). **d**–**f** Show the spatial distribution of pollen-inferred time-series that have a membership degree >0.5 to a particular cluster. **j**–**l** Show the spatial distribution of simulated time-series that have a membership degree >0.5 to a particular cluster

Fuzzy-clustering of derived standardized pollen-based precipitation time-series yielded three clusters with contrasting Holocene precipitation trends (Fig. 1a–c) and rather distinct regional distributions (Fig. 1d–f). Sites in the late-Holocene maximum cluster are mostly located in the southern part of China and along a southeast to northwest oriented band extending from Xinjiang to eastern Mongolia (Fig. 1c, f). All show an increasing precipitation trend during the Holocene. Records from the early-Holocene maximum cluster mostly derive from western parts of the study area, which includes the northeastern Tibetan Plateau, the Altai Mountains (northern Xinjiang and western Mongolia), and Yunnan Province in southern China (Fig. 1a, d). They show a declining precipitation trend during the Holocene. In contrast, records from the mid-Holocene maximum cluster derive from a southwest to northeast oriented band that extends from the southeastern Tibetan Plateau, over north-central China and into northeastern China (Fig. 1b, e).

Our proxy-based observation of three contrasting temporal precipitation patterns, each with a rather distinct spatial extent, contrasts with results from previous investigations of variations in Holocene precipitation levels in China^17–21^. These previous investigations may have been unable to obtain similar results because they suffered from low signal-to-noise ratios resulting from the synthesis of a number of different proxies, from poor spatial coverage, or from more restricted areas of investigation.

It has recently been suggested that strong summer insolation such as occurred during the early or middle Holocene may have led to the northward displacement of the westerly jet stream occurring earlier in the year than it does today, and thus to a prolongation of the monsoonal period in the northern parts of China. In contrast, during periods of low summer insolation, such as occurred during the late Holocene, the EASM would be retained for longer in a transitional position supporting an elevated moisture supply to southern China^6,23,24^. Although this hypothesis may to some extent explain the contrasting Holocene precipitation trends that we interpreted for the northeastern Tibetan Plateau and southern China, it fails to explain the mid-Holocene precipitation maximum reconstructed using the high number of records from the southeastern Tibetan Plateau and from northern and northeastern China. According to this hypothesis, the entire northern part of China should have experienced a decreasing precipitation trend throughout the Holocene, correlating with the reduction in summer insolation, but this is not supported by our pollen-based reconstructions. A prolonged northern position for the westerly jet stream in times of stronger summer insolation cannot alone explain the inferred Holocene precipitation pattern.

### Rainfall patterns as inferred from a minimal empirical model

Modern wind records indicate that the typical zonal orientation of the westerly jet-stream axis regularly changes to a southwest/northeast orientation over eastern Asia^28^. This results in an extreme northerly position for the westerly jet stream, placing it over northeastern China and eastern Mongolia, which is reflected by a characteristic precipitation surplus on the eastern part of the Tibetan Plateau and by a precipitation deficit in northern and north-central China^28^.

We, therefore, hypothesize that contrasting regional moisture patterns, as depicted by the three clusters of pollen-based moisture trends, result from a change in orientation and/or zonal displacement of the westerly jet stream in summer due to a hemisphere-wide reorganization of atmospheric circulation as a result of orbitally driven insolation changes.

**Fig. 2:**
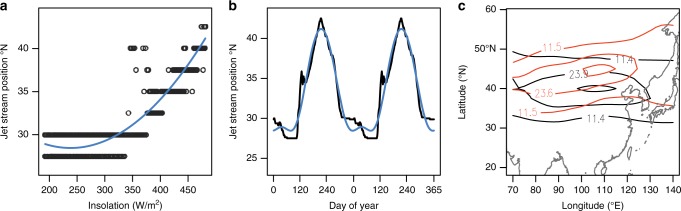
Modern relationship between insolation and the position of the westerly jet stream, and modeled Holocene winds: **a** relationship between the daily climatological position of the westerly jet stream and daily insolation levels (black), and the fitted second-order polynomial (blue); **b** observed (black) and predicted (blue) annual cycles in the position of the westerly jet stream; **c** modeled 250 hPa wind for 2 ka (black) and 9 ka (red) on the day of the year when the westerly wind stream reached its most northerly position, indicating the position and orientation of the westerly jet stream during the early Holocene compared with the late Holocene (for version with wind vectors see Supplementary Fig. 1)

To test this hypothesis we established a minimal empirical model (see the Methods section) that relies solely on the modern relationship between insolation, the position of the westerly jet stream, and precipitation (Fig. 2). A similar model was used successfully to simulate temperature changes on astronomical time-scales^29^. The model estimates empirical transfer functions between daily insolation^30^ and the daily position of the westerly jet stream (within the 100–120°E corridor)^31^ (Fig. 2a, b). Applying these transfer functions to the long-term insolation curve allows us to model past positions of the westerly jet stream. Past precipitation is then estimated for each paleowind position through comparisons with the precipitation patterns of modern analogs (from daily APHRODITE precipitation data covering the 1950–2007 period^32^).

In view of the fact that our minimal empirical model contains only those nonlinearities and feedbacks represented in the observed climate of the last few decades and ignores potential changes in the past moisture source, the similarity of the reconstructions is impressive. The simulated temporal trends (Fig. 1g–i) and spatial patterns obtained for precipitation following cluster analysis (Fig. 1j–l) are mostly in good accordance with those from the pollen-based precipitation reconstructions, including an increase in precipitation during the Holocene in southern China, a band of records with a mid-Holocene maximum that extends from the southeastern Tibetan Plateau via north-central China to northeastern China, and a widespread reduction in precipitation in most of western China and Yunnan Province. This visual inspection is also confirmed by correlation analyses between observed and simulated precipitation time-series. The mean correlation of the raw records (*N* = 101) with the model results is 0.11 (*p* < 0.01). We assume that the low (yet significant) correlation is caused by spatial shifts between the exact location of the trends in the simulated and reconstructed trends (e.g., the northward shift of the decreasing cluster in southern China in the simulation). It could also originate from a number of low-resolution and thus noisy records. This is indicated by a mean correlation of the spatially and temporally smoothed records results of *R* = 0.35 (*p* < 0.01) when accounting for the low-resolution records by calculating the weighted mean of the correlations with the number of observations per record as weight.

**Fig. 3:**
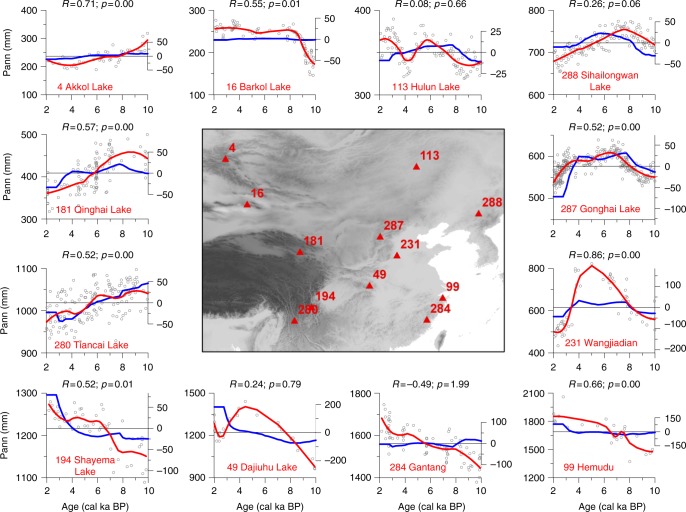
Reconstructed and simulated precipitation of selected sites in China and Mongolia. Pollen-based reconstructions of annual precipitation (mm/year; black circles are original data points, red lines is loess smoothed values (span: 0.5, left scale) compared to simulated precipitation anomalies (mm/year, blue lines, right scale) using a minimal empirical model of selected sites. Correlation coefficient and *p*-values are indicated (i.e., between loess-smoothed reconstructions and simulated time-series from the respective grid-cell). Central map shows the locations of the sites: only sites that had a sufficiently high-resolution and a reliable age model were selected (see Supplementary Table 3 for further details). (Note axes are not on the same scale.)

Comparing selected high-resolution proxy-records with corresponding regional results from the minimal empirical model indicates agreement not only with respect to the major trends in precipitation but also, in most cases, with respect to the magnitude of the changes (Fig. 3). For example, a long-lasting mid-Holocene precipitation maximum of ~100 mm above the average precipitation values in both the early and late Holocene was indicated for the Lake Gonghai record (north-central China) by both approaches. However, model-inferred anomalies from a number of sites are smaller than those in the pollen-based reconstruction. This may be due to the impact of long-term feedback mechanisms (e.g., slow vegetation and soil response to precipitation change) that in the past enhanced the sensitivity of precipitation to variations in insolation, but which are not reflected in modern climatological observations and are therefore not taken into account in our minimal empirical model.

**Fig. 4:**
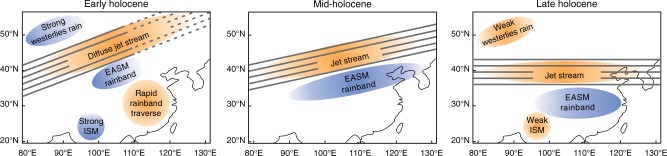
Sketch maps of eastern Asia showing the major summer precipitation and circulation characteristics for early, middle, and late Holocene. Original results were derived by applying pollen-based transfer functions to 101 fossil pollen records and using a minimal empirical model based on present-day relationships between insolation, the position of the westerly jet stream, and spatial distribution patterns for precipitation. (EASM—East Asian Summer Monsoon, ISM—Indian Summer Monsoon)

For the early Holocene our minimal empirical model simulates a southwest to northeast orientation of the westerly jet stream, extending from the Tarim Basin in northwest China to eastern Mongolia (Figs. 2c, 4) and resulting in dry surface conditions (Fig. 1f). The main summer rain band of the EASM, which is always located to the south of the westerly jet stream^5^, is responsible for the reconstructed early-Holocene moisture maximum in the northeastern part of the Tibetan Plateau (Figs. 1d,  4). According to the pollen-based reconstructions (Fig. 1d) and in contrast to the modeled results (Fig. 1j), the rain band did not extend any further to the northeast of the Tibetan Plateau, possibly because of the weakened jet stream in the northern regions (see reconstructions in Fig. 3c and Supplementary Fig. 1 and modern analog circulation pattern^28^), reflecting the weak meridional temperature gradient in the early Holocene. Dry surface conditions in Mongolia and a weak jet stream are also supported by a dust provenance study from the Japan Sea indicating that dust from the remote Taklamakan desert is reduced relative to dust from the nearby Gobi desert^23^. A weak jet stream usually also leads to a weakening of the jet stream-induced adiabatic circulations which may, in turn, reduce the amount of rainfall^31^. Both pollen-based reconstructions and model-based simulations indicate that the early Holocene was rather dry in most parts of southern China (Fig. 1c, i) (a conclusion also supported by paleoclimate simulations using ESMs), probably because the EASM traversed the area so rapidly^24^. Only the most southwesterly part of China (Yunnan Province) received high levels of precipitation, probably carried into the area by an enhanced Indian Summer Monsoon as suggested by analogies to modern circulation^22,33^ and in accordance with evidence from a proxy synthesis from the Indian monsoon region^19,22^.

Despite overall agreement, we also observe certain mismatches between proxy-based and model-based results. Most striking is the difference in the reconstructed precipitation levels for the late Holocene in dry land areas of northwestern China and Mongolia. While pollen-based reconstructions indicate late-Holocene precipitation maxima in these areas, the simulation indicates a decrease since the mid-Holocene. This mismatch likely originates with the pollen-based reconstructions. As has been speculated before, in dry areas pollen assemblages may rather reflect precipitation-to-evaporation (PE) ratios than precipitation alone^18,34^. But additionally, the predictive strength of our model is likely to be less outside the transition area of the monsoonal rain band, such as the northwestern-most part of the study area (in accordance with results of model evaluation), because processes other than westerly-monsoon interactions dominate precipitation generation there.

Site-to-site mismatches may also originate from local or site-specific peculiarities of some of the pollen archives, which can distort the regional-scale representativeness of the respective pollen record because of variations in, for example, the pollen source area and human impact on vegetation.

## Discussion

Our modeling study uses an analog-based approach; i.e., for each day in the past a westerly jet position is modeled (using the insolation-westerly position relationship see Fig. 2a) and then a certain ensemble of analog days is identified using the NCEP/NCAR reanalysis dataset. As this dataset has been explored in many climatological studies^28^ we can speculate on past circulation patterns and teleconnections by making inferences using the modern pattern as an analog.

The observational record from recent decades indicates that reorientation of the westerly jet stream (together with associated changes in the precipitation pattern) tend to occur in years when the North Pacific is relatively warm and when the tropical Pacific and the Indian Ocean are relatively cool, such as during the decay period of a La Niña event^28,35^. The weak zonal insolation gradient during the early Holocene compared to the present gradient correlates with a weaker zonal sea-surface temperature gradient than at present^36^. This may have resulted in the regular occurrence of broad-scale circulation patterns resembling those of a La Niña event over the northern Pacific and adjacent Asian land mass during the early Holocene (or at least a more frequent occurrence of such patterns during the early Holocene than during the middle and late Holocene). However, clear proxy evidence for such an inference is lacking. Furthermore, simulation with an ESM has indicated that remnants of the Laurentide Ice Shield may have intensified the wave structure of the westerly jet stream^37^, thus leading to a marked reorientation of the axis of the westerly jet stream in eastern Asia and the resulting pattern of precipitation.

Our results also suggest that the Holocene decline in Northern Hemisphere summer insolation^30^ caused a southeastward shift in the axis of the westerly jet stream (Fig. 4), thereby relocating the rain band across the Beijing area, which is likely to have caused the mid-Holocene precipitation maximum in that area. By analogy with modern climate records, a longer retention of the westerly jet stream in a transitional position during the summer may result from a frequent development of strong El-Niño events and the associated increase in the sea-surface temperature gradient between the mid-latitudes and the tropical Pacific^28^. This interpretation is in accordance with proxy data, which indicate an increase in the El-Niño–Southern Oscillation (ENSO) variability between the middle and late Holocene^38,39^, as has also been supported by modeling^40^. Previous modeling has indicated a connection between the ENSO and the EASM over long time-scales^10^, and the ENSO may ultimately be attributable to intensified African and Asian monsoons and broad-scale orbitally induced east–west circulation^41^, illustrating the complexity of the eastern hemisphere circulation system. Although our study may provide some hints that low–mid-latitude teleconnections in the eastern hemisphere are rather stable across time-scales it was not set up to prove these teleconnections. There is still much controversy about the Holocene ENSO development, but our study can signal future research directions on the ENSO-EASM relationships.

The consistent spatiotemporal precipitation pattern revealed by our empirical and minimal modeling approaches contradicts results obtained for the Holocene from simulations using ESMs^6,20,42^. Our minimal model is based on the relationship between modern insolation and broad-scale wind fields. The position, orientation, and speed of the westerly jet depend largely on the broad-scale atmospheric temperature pattern. Both are represented well in reanalysis data. Precipitation is inferred from observational data and not taken from the reanalysis. Our simple analog-based simulation appears to reproduce better the proxy-inferred Holocene precipitation trends and quantitative precipitation anomalies than ESMs of far greater complexity. This is because the same mesoscale processes that control the present-day seasonal relationships between the position and orientation of the westerly jet stream and monsoonal precipitation patterns are also likely to have been responsible for the Holocene precipitation pattern. Most of the processes related to the formation of clouds and rainfall work on a subgrid-scale in ESMs and are parameterized. For example, wave activity within the jet (e.g., mesoscalic wave disturbances) causes the small-scale strong air uplift that leads to cloud formation and eventually to precipitation. Surprisingly, retaining these mesoscale relationships appears to be more important for simulating EASM precipitation changes than an accurate representation of broad-scale long-term variability in insolation-driven ocean–atmosphere interactions.

It has been shown that increasing the accuracy of the representation of mesoscale processes in models used to predict precipitation changes in China over the next few decades can even produce a reversal in regional precipitation trends^43^. Achieving an improved representation of the mesoscale circulation processes involved in interactions between the EASM and the westerly jet stream is therefore of utmost importance for a reliable prediction of future precipitation levels in Asia. Pollen-and model-based precipitation reconstructions from this study could be used to validate paleoclimate simulations from such a new generation of models.

## Methods

### Pollen-based reconstructions

This study reconstructs quantitative changes of annual precipitation during the Holocene from a set of fossil pollen records. Our approach^44^ involves the development of a representative modern pollen–precipitation training-set, the set up and evaluation of pollen–precipitation transfer functions that model the relationship between modern pollen occurrences, the application of a transfer function to fossil pollen records to infer past precipitation levels, and the synthesis of spatiotemporal precipitation patterns. Such an approach involves a number of statistical and (paleo-)ecological assumptions^44^ including that precipitation is the major determinant of vegetation and hence pollen composition.

The modern pollen dataset (Source Data File 1) from China and Mongolia used to establish a transfer function between pollen composition and annual precipitation is derived from 2559 sampling sites (gradient: 35–2091 mm) and comprises 161 taxa each being present in at least 3 samples and making up at least 3% of the pollen in at least one sample^45^. The results of a canonical correspondence analysis suggest that, of all the climate variables tested, annual precipitation has the greatest influence on pollen composition within the dataset and this was therefore considered to be the best climate variable to use for reconstructions^25^. It may be that summer monsoon precipitation determines vegetation distribution and not annual precipitation, but our analyses revealed that summer precipitation and annual precipitation are highly correlated and that summer precipitation or precipitation of certain months do not explain better the modern pollen distribution, which instead may be explained by a memory effect of precipitation induced by rain-infiltrated soils (Supplementary Fig. 2 and Supplementary Table 1). A canonical correspondence analysis with a subset of southern China indicated that precipitation is a significant variable for pollen composition also in southern China despite it being more humid (Supplementary Table 2). We selected a unique pollen–precipitation calibration set for each fossil pollen record from modern sites within a 1,000 km radius as Cao and co-workers^45^ have shown that this will increase the reliability of the reconstruction. The geographic distance between each modern sampling site and each fossil pollen record was calculated using the *rdist.earth* function in the *fields* package (version 6.8) for R software^46^.

We used weighted-averaging partial least squares regression (WA-PLS) to set up pollen–precipitation transfer functions. This commonly used method is able to model unimodal responses and it maximizes the information in the modern dataset used for prediction. Exploration of our modern pollen–precipitation dataset revealed that WA-PLS yields more reliable quantitative precipitation reconstructions that the modern analog technique (MAT)^25^—the other common method in paleoenvironmental reconstructions. A pollen–precipitation WA-PLS transfer function was established for each calibration set using the *WAPLS* function in the *rioja* package, version 0.7-3^47^, with leave-one-out cross-validation. The pollen percentages were square-root transformed. The number of WA-PLS components to be employed was selected using a randomization *t*-test^48^.

A taxonomically harmonized fossil pollen dataset from eastern continental Asia^25^ was used to reconstruct past precipitation levels (Source Data File 2). To this dataset, we added 26 new records from areas that previously had poor data coverage. Reconstructions were only made for the period between 10 and 2 cal ka to ensure good spatial coverage and minimize the likelihood of a marked human impact on vegetation^49^. This period was covered by 101 of the available pollen records (details in Supplementary Table 2). A statistical significance test was performed for the quantitative precipitation reconstructions^50^ using the *randomTF* function in the *palaeoSig* package, version 1.1.2^51^. We found that there was no change in the general results if non-significant reconstructions were excluded, and the dataset was therefore not reduced.

Unsupervised fuzzy competitive learning^52^ was used to cluster the precipitation reconstructions and to obtain a degree of membership to each cluster for each reconstruction. Precipitation time-series were smoothed in time (σ = 1000 yrs) and space (σ = 200 km) using a Gaussian kernel. Clustering of smoothed and Z-transformed precipitation reconstructions was implemented with the *cmeans* function (with default values) in the *e1071* package, version 1.6-3^53^. The Xie-Beni index^54^ was calculated to determine the optimal number of clusters in the precipitation reconstruction dataset and to evaluate the validity of clustering partitions, using the *fclustIndex* function in the *e1071* package, version 1.6-3^53^. R code is available in Supplementary Note 1.

### Minimal empirical model

We established a minimal model to simulate the annual precipitation in the area influenced by the EASM and in the EASM-westerly transition area (20–55°N and 80–130°E) over the period between 10 and 2 ka (Source Data File 3). The main driver of Holocene climate changes is change in incoming insolation. Simple empirical models between local daily insolation and local daily temperature have been shown to produce the main features of the Holocene temperature evolution, resembling simulations of state-of-the-art climate models^29^. The jet stream is controlled by a large-scale temperature field^4^, which is also mainly related to insolation. Accordingly, this model assumes that insolation is the first-order control of the jet-stream position and the jet stream position is a major factor in controlling the daily precipitation.

Evidence for the relationship of local insolation and the jet stream position stems from the strong seasonal relationship between the jet-stream position and insolation at 35°N (Fig. 2b) and this relationship is not sensitive to the choice of the latitude of insolation (the *R*^2^ of insolation and jet position is >0.86 for any latitude between 20° and 50°N).

The relationship between the jet stream and precipitation in modern climatology was proposed in several studies^4,5,28^ and confirmed by predicting the local climatological seasonal cycle in precipitation using the jet stream position (Supplementary Fig. 4).

Specifically, we used daily precipitation data from the APHRODITE project (1951–2007)^32^ to obtain a representation of the spatial precipitation patterns. We also used daily u-wind data (i.e., the east–west component of wind) at the 250 hPa pressure level (i.e., the atmospheric height at which the westerly jet stream occurs) from the NCEP reanalysis data (2.5° × 2.5° grid) for the same years as the precipitation data^31^. The daily position of the westerly jet stream was estimated by taking the zonal mean of the u-wind field between 100°E and 120°E and choosing the latitude with the maximum daily windspeed. We parametrized the position of the westerly jet stream as a function of insolation^29^. A second-order polynomial transfer function was fitted between the modern daily insolation at 35°N and the climatological position of the westerly jet stream, after including a time lag of 45 days (= the time lag that maximized the correlation) to take into account the thermal inertia. Our result is not sensitive to the choice of any insolation latitude inside our prediction area. The second-order polynomial resulted in a coefficient of determination (*R*^2^) of 0.88 (compared to an *R*^2^ of 0.78 in a linear model). Past positions of the westerly jet stream were estimated by applying this transfer function to the daily insolation values for the period between 10 and 2 ka^30^. In order to preserve the fine-scale features and the absolute range of the wind positions, the jet-stream position anomalies between the observed and modeled modern jet climatology were added to the reconstructed historical jet-stream positions. The modeled position of the westerly jet stream for a particular day in the past was used to identify the past spatial precipitation pattern. For that purpose, all days (= analog days) in the modern dataset (1951–2007, ~20,000 days) with a westerly jet stream position within ± 5 degrees of latitude of the modeled jet stream position were chosen. We restricted our selection to days within the same season as the targeted historical day (i.e., to ± 60 days) in order to avoid a mixture of spring and autumn precipitation patterns. Finally, the precipitation patterns for the analog days in the modern dataset were then averaged to obtain an average precipitation pattern for each day, and annual means were calculated. To estimate the wind-field corresponding to the jet-stream position and precipitation patterns (Fig. 2), we averaged the analog days in the modern u-wind and v-wind datasets to obtain the average wind field for each day. R code is available in Supplementary Note 1.

### Correlation analyses

We performed correlation analyses between time-series of pollen-based precipitation reconstruction and simulated precipitation. In a first analysis (Fig. 3), the model results are linearly interpolated to the sample times of the reconstructed record. We account for the autocorrelation in the significance estimates by adjusting the effective degrees of freedom using the estimated lag-1 correlation of the reconstructed records. Second, the mean correlation of the spatially and temporally smoothed records was investigated. To account for the temporal autocorrelation, as well as the effect of kernel smoothing in space and time on the temporal and spatial correlation structure, we perform a Monte-Carlo experiment. We create a set of surrogate records by simulating a random annual time-series, following a power-law scaling with beta = 1 and block averaging the record to the sampling resolution of the true record. In analog to the observed records, this set of surrogate records is then smoothed in time and space using the Gaussian kernel and the (weighted) correlation to the model results is estimated. Repeating this 1000 times allows an empirical estimation of the *p*-value. R code is available in Supplementary Note 1.

## Supplementary information

Supplementary Information

Supplementary Software

Source Data

## Data Availability

The taxonomically harmonized modern and fossil pollen datasets used for this study as well as the reconstructed and simulated P_ann_ time-series are attached as source data files (Source Data).

## Code availability

R code for pollen-based climate reconstructions as well as clustering of pollen-based and simulated time-series are stored in Supplementary Note 1. Code for the minimal empirical model can be accessed from [https://github.com/EarthSystemDiagnostics/MonsoonMinimalModel].
