# Supplementary material for: Position and orientation of the westerly jet determined Holocene rainfall patterns in China
Source: Nat Commun. 2019 May 30;10:2376. doi: 10.1038/s41467-019-09866-8 (PMC6542844; doi:10.1038/s41467-019-09866-8)

Supplementary Material

Herzschuh et al., 2019

Position and orientation of the westerly jet determined Holocene rainfall patterns in China
Nature Communications

Supplementary Tables

Supplementary Tab. 1 Summary statistics for canonical correspondence analyses for the whole dataset from China and Mongolia (Cao et al., 2014). P_{ann} – annual precipitation, Mt_{wa} – mean temperature of the warmest month; Mt_{co} – mean temperature of the coldest month; T_{ann} mean annual temperature, P_{amjjas} – precipitation between March and September, P_{amjjas} – precipitation between June and August. Results indicate that P_{ann} explains most variance in the modern pollen dataset. Neither temperature nor seasonal precipitation explains more variance.

Climatic variables	VIF (without T_{ann})	VIF (add T_{ann})	λ_1/λ_2	Climatic variables as sole predictor		Marginal contribution based on climatic variables	
				Explained variance (%)	P-value	Explained variance (%)	P-value
P_{ann}	3.8	3.8	1.58	4.9	0.005	1.50	0.005
Mt_{co}	4.3	221.7	1.36	4.2	0.005	0.67	0.005
Mt_{wa}	1.5	116.6	0.61	2.7	0.005	1.30	0.005
T_{ann}	-	520.4	-	-		-	-
Pamjjas	-	-	1.50	4.9	0.005		
Pjja	-	-	1.30	4.4	0.005		

Cao, X., Herzschuh, U., Telford, R.J., Ni, J. A modern pollen-climate dataset from China and Mongolia: assessing its potential for climate reconstruction. Review of Palaeobotany and Palynology 211, 87-96 (2014).

Supplementary Tab. 2 Summary statistics for canonical correspondence analyses for the southern China dataset <30°N (Cao et al., 2014). P_{ann} – annual precipitation, Mt_{wa} – mean temperature of the warmest month; Mt_{co} – mean temperature of the coldest month; T_{ann} mean annual temperature. Results indicate that P_{ann} explains most variance in the modern pollen dataset. Neither temperature nor seasonal precipitation explains more variance. Results indicate that even in the southern part of China precipitation is the variable that explains most variance in the modern pollen dataset.

Climatic variables	VIF (without T_{ann})	VIF (add T_{ann})	λ_1/λ_2	Climatic variables as sole predictor		Marginal contribution based on climatic variables	
				Explained variance (%)	P-value	Explained variance (%)	P-value
P_{ann}	2.5	2.6	0.89	4.5	0.001	1.82	0.001
Mt_{co}	5.0	400.1	0.83	4.1	0.001	1.45	0.001
Mt_{wa}	4.5	342.5	0.80	4.4	0.001	1.50	0.001
T_{ann}	-	1355.9	-	-		-	-

Supplementary Tab. 3. Supplementary information for each pollen record. We assessed the age-model reliability (age score) and pollen data quality (data score) of each record to be high, intermediate, or low. An age-model was considered highly reliable if it had more than 3 reliable dates within the 10–2 cal ka BP interval, and was considered to be of low reliability if it had only one reliable date, or no dating at all, within that interval. The data quality was considered to be high if it possessed a complete pollen assemblage and original pollen data. The component (Comp) means the selected component of the WA-PLS model; r^2 is the coefficient of determination between observed and predicted environmental values; RMSEP is the root mean square error of prediction; RMSEP percentage means the RMSEP as a percentage of the modern annual precipitation gradient range (P_{ann} gradient); Sig. is the p-value of the statistical significance test of reconstruction (Telford & Birks, 2011).

Site information						Quality of pollen data			Reliability of reconstruction							Reference
ID	Site	Long.	Lat.	Alt.	Modern Pann	Age score	Data score	Available number	No. of Sample	Pann gradient	Comp.	r^2	RMSEP	RMSEP percentage	Sig.	
1	Achit Nur Lake	90.60	49.50	1435	273	2	2	11	292	60-541	2	0.67	80	16.67	0.01	Gunin et al., 1999
4	Akkol Lake	89.63	50.25	2204	411	3	3	44	283	60-541	2	0.67	81	16.84	0.04	Blyakharchuk et al., 2007
7	Ayongwama Co	98.20	34.83	4220	357	2	1	17	944	35-1069	1	0.79	115	11.14	0.04	Cheng et al., 2004
10	Baikal Lake	105.87	52.08	130	2401	3	3	29	164	93-378	1	0.47	49	17.05	0.01	Demske et al., 2005
16	Barkol Lake	92.80	43.62	1575	163	3	2	85	471	35-541	1	0.63	90	17.84	0.06	Tao et al., 2009
17	Bayanchagan Lake	115.21	41.65	1355	389	3	2	30	584	111-864	1	0.56	115	15.24	0.25	Jiang et al., 2006
22	Bosten Lake	86.55	41.97	1050	74	3	3	18	296	47-541	1	0.68	91	18.48	0.95	Xu, 1998
24	Bunan Lake	90.83	35.95	4876	217	1	2	4	697	40-764	1	0.66	94	12.98	0.16	Shan et al., 1996
28	Chaiwopu Lake	87.78	43.55	1100	223	1	2	6	254	35-541	1	0.70	89	17.64	0.71	Li and Yan, 1990
29	Changjiang River_1997	121.38	31.62	2	1109	3	3	9	249	488-1913	2	0.81	185	12.97	0.53	Yi et al., 2003
30	Changjiang River_1998	120.23	32.25	6	1059	3	3	44	356	476-1913	2	0.81	178	12.42	0.74	Yi et al., 2003
41	Daba Nur Lake	98.79	48.20	2465	365	3	2	12	309	35-541	1	0.67	78	15.39	0.55	Gunin et al., 1999
42	Dabsan Lake	95.50	37.10	2675	58	2	2	4	931	35-1062	1	0.68	115	11.15	0.64	Du and Kong, 1986
45	Dahaizi Lake	102.67	27.83	3660	1069	3	2	17	654	312-1838	1	0.78	180	11.81	0.59	Li and Liu, 1988
46	Dahu Lake	115.03	24.25	250	1780	3	2	15	576	1020-2091	2	0.60	195	18.20	0.37	Zhou et al., 2004
47	Daihai Lake_2004	112.67	40.58	1220	376	3	3	192	643	35-1129	1	0.62	117	10.67	0.05	Xiao et al., 2004
49	Dajiuhu Lake	110.67	31.75	1700	1130	3	2	25	668	144-2011	1	0.81	213	11.39	0.01	Liu H.P. et al., 2001
50	Dalai Nur Lake	116.58	43.28	1200	349	1	2	3	591	111-1006	2	0.66	102	11.43	0.2	Li et al., 1990
52	Daluoba	88.20	47.83	2020	209	1	2	5	252	60-541	2	0.70	81	16.90	0.08	Yan and Xu, 1992
58	Dengjiacun	113.65	34.47	133	661	3	1	9	615	144-1857	2	0.83	159	9.29	0.93	Zhang et al., 2007
61	Dingnan	115.03	24.75	274	1683	3	2	13	579	1020-2091	2	0.61	195	18.17	0.05	Dodson et al., 2006
64	Dongganchi	115.78	39.53	49	509	3	3	42	573	111-1325	1	0.80	115	9.46	0.4	Zhang et al., 1997

66	Dood Nur Lake	99.38	51.33	1538	334	2	2	8	187	37-378	1	0.44	57	16.79	0.01	Gunin et al., 1999
68	Dunde	96.40	38.10	5325	339	3	3	24	944	35-872	1	0.67	111	13.25	0.66	Liu et al., 1998
69	Ebinur Lake	82.45	44.55	212	174	3	2	7	235	53-541	2	0.76	81	16.70	0.01	Wen and Qiao, 1990
71	Erhai Lake	100.20	25.77	1974	785	3	1	40	462	403-1766	1	0.81	152	11.14	0.74	Zhou et al., 2003
72	Erhailongwan Lake	126.37	42.30	724	813	3	2	8	373	220-1006	1	0.60	94	11.94	0.47	Liu et al., 2008
78	Ganhai Lake	112.19	38.89	1854	505	3	3	21	714	40-1348	2	0.81	110	8.44	0.16	Meng et al., 2007
81	Gladkoye Bog	83.33	55.00	80	458	3	3	45	45	96-271	1	0.00	35	19.80	0.68	Firsov et al., 1982
82	Gounong Co	92.15	34.63	4670	274	3	3	31	722	47-872	1	0.66	108	13.15	0.02	Shan et al., 1996
83	Grusha Lake	89.42	50.38	2413	336	3	3	13	270	60-541	2	0.68	82	16.99	0.04	Blyakharchuk et al., 2007
84	Guangfulin	121.19	31.06	4	1179	2	1	35	296	490-1913	2	0.80	177	12.42	0.59	Atahan et al., 2008
87	Gucheng Lake	118.90	31.28	6	1273	2	3	153	371	476-2089	2	0.81	197	12.21	0.87	Yang et al., 1996
88	Gun Nur Lake	106.60	50.25	600	377	3	3	21	252	35-402	1	0.49	57	15.64	0.1	Gunin et al., 1999
91	Halali	99.78	36.72	3220	360	3	2	2	853	35-1062	1	0.74	116	11.30	1	Chen F.H. et al., 1991
95	Haoluku Lake	116.76	42.96	1295	382	2	3	24	595	111-1006	2	0.66	102	11.42	0.87	Wang et al., 2001
99	Hemudu	121.35	29.97	6	1412	3	3	23	244	537-2068	2	0.76	180	11.74	0.36	Li et al., 2009
104	Hongyuanbai River	102.53	32.80	3500	772	3	3	33	935	40-1456	2	0.85	122	8.59	0.06	Wang, 1987
107	Huangjiapu	115.15	40.57	500	376	1	1	16	541	111-1051	1	0.62	117	12.42	0.73	Sun et al., 2001
108	Huangsha	113.23	23.13	40	1822	2	2	16	541	1016-2091	2	0.59	193	17.96	0.83	Li, 1991
111	Huguangyan Maar Lake	110.28	21.15	88	1690	3	3	4	429	892-2091	1	0.50	201	16.76	0.59	Lv et al., 2003
113	Hulun Nur Lake_2006	117.51	49.13	545	284	3	2	103	427	111-710	1	0.49	93	15.49	0.77	Wen et al., 2010
114	Hurleg Lake	96.90	37.28	2817	117	3	3	43	857	35-1062	1	0.71	115	11.16	0.4	Zhao Y. et al., 2007
119	Jiangjunpaozi Lake	117.47	42.37	1490	480	2	1	8	566	111-1006	2	0.66	103	11.56	0.24	Liu H.Y. et al., 2001
124	Jiudaogou	96.65	40.50	2143	59	2	1	24	841	35-806	1	0.59	116	15.06	0.87	Mao et al., 2007
126	Juyan Lake	101.85	41.89	892	40	3	3	59	796	35-850	1	0.64	110	13.46	0.48	Herzschuh et al., 2004
130	Kendegelukol Lake	87.64	50.51	2050	348	3	3	25	247	60-541	2	0.71	81	16.91	0.02	Blyakharchuk et al., 2004
134	Kotokel Lake	108.12	52.78	458	348	3	2	58	210	111-492	1	0.77	46	12.02	0.91	Tarasov et al., 2009
138	Kucha Lake	97.24	34.01	4540	481	3	3	38	929	35-1091	1	0.78	118	11.21	0.1	Herzschuh et al., 2009
139	Kuhai Lake	99.31	35.52	4150	427	3	3	32	941	35-1069	1	0.79	114	11.02	0.51	Wischnewski et al., 2011
144	Liuzhouwan Lake	116.68	42.71	1365	398	2	2	14	594	111-1006	2	0.65	104	11.58	0.21	Wang et al., 2001
146	Longquan Lake	112.24	31.08	80	990	2	2	21	667	179-2091	2	0.86	211	11.03	0.73	Liu, 1991
147	Lop Nur_1983	90.25	40.33	800	17	1	2	7	530	35-541	1	0.63	90	17.83	0.1	Yan et al., 1983
149	Luanhaizi Lake	101.35	37.59	3200	485	3	3	9	763	35-1062	1	0.75	117	11.37	0.38	Herzschuh et al., 2005
151	Luojiang	121.36	29.98	4	1406	2	2	8	241	537-2011	2	0.75	178	12.08	0.1	Atahan et al., 2008
153	Manas Lake	85.92	45.83	251	125	3	3	31	230	47-541	2	0.74	81	16.39	0.31	Sun et al., 1994
159	Mengcun	117.12	38.05	5	552	2	3	19	617	111-1755	2	0.82	140	8.52	0.02	Xu et al., 1993
162	Naleng Co Lake	99.76	31.11	4200	705	3	3	69	922	51-1580	2	0.81	118	7.73	0.15	Kramer et al., 2010
164	Nantun	104.23	26.70	2197	979	3	1	40	564	441-1883	1	0.80	184	12.80	0.64	Chen P.Y. et al., 1991

167	Niangziguan	113.88	37.95	500	519	1	2	5	638	104-1755	2	0.81	145	8.79	0.35	Yang et al., 1999
179	Qidong	121.70	31.90	10	1089	3	3	16	249	488-1880	2	0.82	182	13.06	0.23	Liu et al., 1992
181	Qinghai Lake	100.52	36.67	3200	421	3	3	114	816	35-1062	1	0.75	117	11.41	0.01	Liu et al., 2002
183	Qiumu	99.87	26.53	2200	916	1	3	10	568	338-1766	2	0.81	136	9.49	0.8	Kong et al., 1986
184	Qongjiamong Co	92.37	29.81	4980	556	3	3	35	577	51-1091	1	0.75	113	10.85	0.38	Shen, 2003
185	Ren Co	96.68	30.73	4450	653	3	2	21	844	51-1192	2	0.79	113	9.93	0.38	Tang et al., 1999
189	Sanyixiang	117.38	43.62	1541	417	2	1	6	582	111-1006	2	0.66	102	11.43	0.46	Wang et al., 2005
190	Selin Co	88.52	31.57	4530	299	3	3	30	364	51-743	1	0.75	84	12.18	0.11	Sun et al., 1993
194	Shayema Lake	102.22	28.58	2400	1034	3	3	53	730	150-1838	1	0.80	174	10.34	0.01	Tang and Shen., 1996
195	Shidicun	117.52	43.25	1077	390	2	2	4	580	111-1006	2	0.66	102	11.43	0.23	Liu, 2002
206	Sujiawan	104.52	35.54	1950	426	3	3	32	861	35-1348	2	0.86	109	8.28	0.01	Feng et al., 2006
219	Tolmachevsko	84.00	55.00	110	715	3	3	18	49	89-271	1	0.04	41	22.67	0.75	Volkov and Arkhipov, 1978
225	Ulan Ul Lake	90.50	34.87	4854	249	1	2	8	604	47-764	1	0.67	93	12.94	0.23	Shan et al., 1996
227	Uzunkol Lake	87.11	50.48	1985	168	3	2	47	242	60-541	2	0.71	82	16.99	0.54	Blyakharchuk et al., 2004
231	Wangjiadian	114.67	36.16	67	532	3	3	54	622	137-1857	2	0.81	157	9.11	0.05	Cao et al., 2010
237	Wuqia Bridge	83.50	43.20	1320	410	1	2	4	250	53-541	2	0.75	82	16.76	0.66	Lin, 1994
238	Wuying River	105.82	35.76	1400	435	3	3	32	892	35-1348	2	0.86	108	8.23	0.49	Xia et al., 1998
244	Xiehu Lake	121.00	37.38	0	711	2	2	22	415	208-1755	1	0.77	140	9.04	0.27	Zhou et al., 2008
245	Ximen Co Lake	101.47	33.38	4020	720	3	3	34	1003	35-1348	2	0.85	118	8.96	0.14	Herzschuh et al., 2014
246	Xinghua	119.88	32.71	2	1031	2	2	12	385	376-1869	2	0.83	173	11.61	0.06	Shu et al., 2008
249	Yamant Nur Lake	102.60	49.90	1000	179	1	2	23	212	35-378	2	0.70	49	14.40	0.01	Gunin et al., 1999
250	Yangerzhuang	117.35	38.35	5	562	2	3	21	583	111-1755	2	0.83	133	8.12	0.01	Xu et al., 1993
251	Yanghu Lake	84.65	35.43	4778	189	2	2	10	386	51-680	1	0.67	88	14.05	0.14	Zhao Z.M. et al., 2007
255	Yangyuan_Xipu	114.22	40.12	912	389	2	1	2	549	53-1129	1	0.65	118	10.93	0.13	Wang et al., 2003
258	Yidun Lake	99.55	30.30	4470	726	2	2	15	902	51-1597	2	0.83	118	7.63	0.6	Shen et al., 2006
268	Zigetang Lake	90.90	32.00	4560	384	3	3	46	519	51-832	1	0.74	89	11.42	0.21	Herzschuh et al., 2006
270	Zoige_RH	103.35	33.95	3400	655	2	2	15	914	35-1348	2	0.86	119	9.04	0.47	Tang and Shen, 1996
271	Zoige_RM	102.35	33.95	3401	669	3	3	35	949	35-1348	2	0.85	118	9.01	0.26	Shen et al., 1996
272	Dongi Cona Lake	98.50	35.50	4090	311	3	3	33	936	35-1069	1	0.79	114	11.00	0.48	Wang et al., 2014
273	Sumxi Lake	80.24	34.61	5059	68	3	3	70	92	53-433	1	0.75	57	14.89	0.23	Campo and Gasse, 1993
274	Bayan Nuur Lake	93.00	50.00	932	172	3	3	40	311	37-541	2	0.66	80	15.86	0.11	Krengel, 2000
278	Sayram Lake	81.09	44.34	2125	254	3	3	96	224	53-541	2	0.76	81	16.50	0.68	Jiang et al., 2013
279	Qigai Nuur Lake	109.50	39.50	1408	393	3	3	98	882	35-1348	2	0.80	112	8.52	0.01	Sun and Feng, 2013
280	Tiancai Lake	99.72	26.63	3898	970	3	3	154	569	338-1766	2	0.81	131	9.20	0.49	Xiao et al., 2014
281	Shaamar	105.20	50.20	650	291	3	3	16	225	35-378	2	0.56	53	15.52	0.18	Ma et al., 2013
282	Toushe_2013	120.88	23.82	1014	1424	3	3	72	341	1046-2091	2	0.70	159	15.24	0.51	Li H.C. et al., 2013
284	Gantang	119.03	26.77	1007	1900	3	3	85	464	691-2091	2	0.68	198	14.12	0.01	Yue et al., 2012
285	Dajiuhu_2013	110.00	31.49	1751	1130	3	3	27	737	138-2091	1	0.82	211	10.80	0.71	Li J. et al., 2013

287	Gonghai Lake	112.23	38.90	1860	505	3	3	288	704	51-1348	2	0.81	111	8.56	0.44	Chen et al., 2015
288	Sihailongwan Lake	126.60	42.28	797	775	3	3	118	366	226-1006	1	0.60	94	12.02	0.32	Stebich et al., 2015
290	Aibi Lake	82.83	45.02	200	95	3	2	51	216	53-541	2	0.76	82	16.74	0.6	Wang et al., 2013
297	Zoige_2011	102.63	33.45	3467	648	3	3	104	943	35-1348	2	0.85	119	9.08	0.25	Zhao et al., 2011

References Supplementary Tab. 1

Atahan, P., Itzstein-Davey, F., Taylor, D., Dodson, J., Qin, J., Zheng, H., Brooks, A., 2008. Holocene-aged sedimentary records of environmental changes and early agriculture in the lower Yangtze, China. Quaternary Science Reviews, 27, 556-570.

Blyakharchuk, T.A., Wright, H.E., Borodavko, P.S., van der Knaap, W.O., Ammann, B., 2007. Late Glacial and Holocene vegetational history of the Altai Mountains (southwestern Tuva Republic Siberia). Palaeogeography, Palaeoclimatology, Palaeoecology 245, 518-534.

Blyakharchuk, T.A., Wright, H.E., Borodavko, P.S., van der Knaap, W.O., Ammann, B., 2004. Late Glacial and Holocene vegetational changes on the Ulagan high-mountain plateau, Altai Mountains, southern Siberia. Palaeogeography, Palaeoclimatology, Palaeoecology 209, 259-279.

Campo, E.V., Gasse, F., 1993. Pollen- and diatom-inferred climatic and hydrological changes in Sumxi Co Basin (Western Tibet) since 13,000 yr B.P. Quaternary Research 39, 300–313.

Cao, X., Xu, Q., Jing, Z., Tang, J., Li, Y., Tian, F., 2010. Holocene climate change and human impacts implied from the pollen records in Anyang, central China. Quaternary International 227, 3-9.

Chen, F., Xu,Q., Chen, J., Birks, H.J.B., Liu, J., Zhang, S., Jin, L., An, C., Telford, R.J., Cao, X., Wang, Z., Zhang, X., Selvaraj, K., Lu, H., Li, Y., Zheng, Z., Wang, H., Zhou, A., Dong, G., Zhang, J., Huang, X., Bloemendal, J., Rao, Z., 2015. East Asian summer monsoon precipitation variability since the last deglaciation. Scientific Reports 5, 11186. doi: 10.1038/srep11186.

Chen, F.H., Wang, S.L., Zhang, W.X., Pan, B.T., 1991. The loess profile at south bank, climatic information and lake-level fluctuations of Qinghai Lake during the Holocene. Scientia Geographica Sinica 11, 76-85 (in Chinese with English abstract).

Chen, P.Y., Zhou, Q.Y., Lin, S.J., 1991. Palaeoenvironment from 15000 a B.P. to the present in Weining County, Guizhou: A study of Nantun peat-bed section. Geology of Guizhou 8, 141-152 (in Chinese with English abstract).

Cheng, J., Zhang, X.J., Tian, M.Z., Tang, D.X., Yu, W.Y., Yu, J.K., Qiao, G.B., Zan, LH., 2004. Climate of the Holocene megathermal in the source area of the Yellow River, Northeast Tibet. Geological Review 50, 330-337 (in Chinese with English abstract).

Demske, D., Heumann, G., Granoszewski, W., Nita, M., Mamakowa, K., Tarasov, P.E., Oberhansli, H., 2005. Late glacial and Holocene vegetation and regional climate variability evidenced in high-resolution pollen records from Lake Baikal. Global and Planetary Change 46, 255-279.

Dodson, J.R., Hickson, S., Khoo, R., Li, X.Q., Toia, J., Zhou, W.J., 2006. Vegetation and environment history for the past 14 000 yrBP from Dingnan, Jiangxi Province, South China. Journal of Integrative Plant Biology 48, 1018-1027.

Du, N.Q., Kong, Z.C., 1986. Spore-pollen assemblages of Dabsan Lacustrine sediment CK1/81 and their geographical and ecological significances. In: Qinghai Institute of Salt Lakes, CAS (Ed.), Geological Environmental Changes in Qaidam Basin, Qinghai Province in Late Cenozoic. Science Press, Beijing, pp. 59-70 (in Chinese).

Feng, Z.D., Tang, L.Y., Wang, H.B., Ma, Y.Z., Liu, K.-B., 2006. Holocene vegetation variations and the associated environmental changes in the western part of the Chinese Loess Plateau. Palaeogeography, Palaeoclimatiology, Palaeoecology 241, 440-456.

Firsov, L.V., Volkova, V.S., Levina, T.P., Nikolayeva, I.V., Orlova, L.A., Panychev, V.A., Volkov, I.A., 1982. The stratigraphy, geochronology, and standard spore-pollen diagram for Holocene peat, Gladkoye Bog, Novosibirsk. In: Arkhipov, S.A. (Ed.), Problems of stratigraphy and paleogeography of the Pleistocene of Siberia. Nauka, Novosibirsk. pp. 96-107 (in Russian).

Gunin, P.D., Vostokova, E.A., Dorofeyuk, N.I., Tarasov, P.E., Black, C.C., 1999. Vegetation Dynamics of Mongolia. Kluwer Academic Publishers, London.

Herzschuh, U., Borkowski, J., Schewe, J., Mischke, S., Tian, F., 2014. Moisture-advection feedback supports strong early-to-mid Holocene monsoon climate on the eastern Tibetan Plateau as inferred from a pollen-based reconstruction. Palaeogeography Palaeoclimatology Palaeoecology 402, 44-54.

Herzschuh, U., Kramer, A., Mischke, S., Zhang, C.J., 2009. Quantitative climate and vegetation trends since the late glacial on the northeastern Tibetan Plateau deduced from Koucha Lake pollen spectra. Quaternary Research, 71, 162-171.

Herzschuh, U., Tarasov, P., Wünnemann, B., Hartmann, K., 2004. Holocene vegetation and climate of the Alashan Plateau, NW China, reconstructed from Pollen data. Palaeogeography, Palaeoclimatology, Palaeoecology 211, 1-17.

Herzschuh, U., Winter, K., Wunnemann, B., Li, S.J., 2006. A general cooling trend on the central Tibetan Plateau throughout the Holocene recorded by the Lake Zigetang pollen spectra. Quaternary International 154-155, 113-121.

Herzschuh, U., Zhang, C.J., Mischke, S., Herzschuh, R., Mohammadi, F., Mingram, B., Kurschner, H., Riedel, F., 2005. A late Quaternary lake record from the Qilian Mountains (NW China): evolution of the primary production and the water depth reconstructed from macrofossil, pollen, biomarker, and isotope data. Global and Planetary Change 46, 361-379.

Jiang, Q.F., Ji, J.F., Shen, J., Matsumoto, R., Tong, G.B., Qian, P., Ren, X.M., Yan, D.Z., 2013. Holocene vegetational and climatic variation in westerlydominated areas of Central Asia inferred from the Sayram Lake in northern Xinjiang, China. Science China Earth Sciences 56, 339-353.

Jiang, W.Y., Guo, Z.T., Sun, X.J., Wu, H.B., Chu, G.Q., Yuan, B.Y., Hatte, C., Guiot, J., 2006. Reconstruction of climate and vegetation changes of Lake Bayanchagan (Inner Mongolia): Holocene variability of the East Asian monsoon. Quaternary Research 65, 411-420.

Kong, Z.C., Du, N.Q., Gao, X.L., Huang, X.G., Lin, L.R., 1986. Palynological analysis of the Qiumushan region in Jianchuan County of Holocene and its significance. In: The Comprehensive Scientific Expedition to the Tibet Plateau, the Chinese Academy of Sciences (Ed.), Studies in Tibet Plateau, Special Issue of Hengduan Mountains Scientific Expedition (II). Beijing Science and Technology Press, Beijing, pp. 80-85 (in Chinese).

Kramer, A., Herzschuh, U., Mischke, S., Zhang C.J., 2010. Holocene treeline shifts and monsoon variability in the Hengduan Mountains (southeastern Tibetan Plateau), implications from palynological investigations. Palaeogeography, Palaeoclimatology, Palaeoecology 286, 23-41.

Krengel, M., 2000. Discourse on history of vegetation and climate in Mongolia-palynological report of sediment core Bayan Nuur I (NW-Mongolia). In Walther, M., Janzen, J., Riedel, F., and Keupp, H. (eds). State and dynamics of geosciences and human geography in Mongolia: extended abstracts of the international symposium (Berliner Geowissenschaftliche Abhandlungen). pp. 80-84, Berlin, Germany.

Li, C.H., Tang, L.Y., Wan, H.W., Yao, S.C., Zhang, D.F., 2009. Vegetation and human activity in Yuyao (Zhejiang Province) inferred from the sporo-pollen record since the late Pleistocene. Acta Micropalaeontologica Sinica 26, 48-56 (in Chinese with English abstract).

Li, H.C., Liew, P.M., Seki, O., Kuo, T.S., Kawamura, K., Wang, L.C., Lee, T.Q., 2013. Paleoclimate variability in central Taiwan during the past 30 Kyrs reflected by pollen, δ13CTOC, and n-alkane-δD records in a peat sequence from Toushe Basin. Journal of Asian Earth Sciences 69, 166-176.

Li, J., Zheng, Z., Huang, K., Yang, S., Chase, B., Valsecchi, V., Carré, M., Cheddadi, R., 2013. Vegetation changes during the past 40,000 years in Central China from a long fossil record. Quaternary International 310, 221-226.

Li, R.Q., Zheng, L.M., Zhu, G.R., 1990. Lakes and Environmental Change in the Inner Mongolian Plateau. Beijing Normal University Press, Beijing (in Chinese).

Li, S.J., Zheng, B.X., Jiao, K.Q., 1991. Preliminary research on lacustrine deposit and lake evolution on the south slope of the west Kunlun Mountains. Scientia Geographica Sinica 11, 306-314 (in Chinese with English abstract).

Li, W.Y., Yan, S., 1990. Quaternary spore and pollen research in Chaiwopu Basin. In: Shi,Y.F., Wen, Q.Z., Qu, Y.G., et al. (Ed.), The Quaternary Climo-Environment Changes and Hydrogeological Condition of Chaiwopu Basin In Xinjiang Region. China Ocean Press, Beijing, pp. 164 (in Chinese).

Li, X., Liu, J.L., 1988. Holocene vegetational and environmental changes at Mt. Luoji, Sichuan. Acta Geographica Sinica 43, 44-51 (in Chinese with English abstract).

Lin, M.C., 1994. Spore-pollen analysis of Quaternary in Xinjiang Region. In: Wen, Q.Z. (Ed.), Quaternary Geology and Environment of Xinjiang Region, China. Agricultural Press of China, Beijing, pp. 68-94 (in Chinese).

Liu, G.X., 1991. Late-Glacial and Postglacial vegetation and association environment in Jianghan Plain. Acta Botanica Sinica 33, 581-588 (in Chinese with English abstract).

Liu, H.P., Tang, X.C., Sun, D.H., Wang, K.F., 2001. Palynofloras of the Dajiuhu Basin in Shennongjia Mountains during the last 12.5ka. Acta Micropalaeontologica Sinica 18, 101-109 (in Chinese with English abstract).

Liu, H.Y., Cui, H.T., Huang, Y.M., 2001. Detecting Holocene movements of the woodland-steppe ecotone in northern China using discriminant analysis. Journal of Quaternary Science 16, 237-244.

Liu, K.B., Sun, S., Jiang, X.H., 1992. Environmental change in the Yangtze River Delta since 12000 years B.P. Quaternary Research 38, 32-35.

Liu, K.B., Yao, Z.J., Thompson, L.G., 1998. A pollen record of Holocene climatic changes from the Dunde ice cap, Qinghai-Tibetan Plateau. Geology 26, 135-138.

Liu, X.H., 2002. Study on Holocene environment of Keshiketeng County, Inner Mongolia (Master Thesis). China Geology University, Beijing (in Chinese with English abstract).

Liu, X.Q., Shen, J., Wang, S.M., Yang, X.D., Tong, G.B. and Zhang, E.L., 2002. A 16000-year pollen record of Qinghai Lake and its paleoclimate and paleoenvironment. Chinese Science Bulletin 47, 1931–1936.

Liu, Y.Y., Zhang, S.Q., Liu, J.Q., You, H.T., Han, J.T., 2008. Vegetation and environment history of Erlongwan Maar Lake during the late Pleistocene on pollen record. Acta Micropalaeontologica Sinica 25, 274-280 (in Chinese with English abstract).

Lv, H.Y., Liu, J.Q., Chu, G.Q., Gu, Z.Y., Negendank, J., Schettler, G., Mingram, J., 2003. A study on pollen and environment in the Huguangyan Maar Lake since the last glaciation. Acta Palaeontologica Sinica 42, 284-291 (in Chinese with English abstract).

Ma, Y., Liu, K.B., Feng, Z., Meng, H., Sang, Y., Wang, W., Zhang, H., 2013. Vegetation changes and associated climate variations during the past ~38,000 years reconstructed from the Shaamar eolian-paleosol section, northern Mongolia. Quaternary International 311, 25-35.

Mao, H.L., Zhao, H., Lu, Y.C., Wang, C.M., Zhang, K.Q., Yang, Z.J., Liang, J.J., 2007. Pollen assemblages and environment evolution in Shule River alluvial fan oasis of Gansu. Acta Geoscientica Sinica 28, 528-534 (in Chinese with English abstract).

Meng, X.M., Zhu, D.G., Shao, Z.G., Han, J.E., Yu, J., Meng, Q.W., Lv, R.P., Luo, P., 2007. Paleoclimatic and plaeoenvironmental evolution since Holocene in the Ningwu Area, Shanxi Province. Acta Geologica Sinica 81, 316-323 (in Chinese with English abstract).

Shan, F.S., Kong, Z.C., Du, N.Q., 1996. Palaeovegetation and environmental changes. In: Li, B.Y. (Ed.), Physical Environment of Hoh Xil Region, Qinghai. Science Press, Beijing, pp. 197-206 (in Chinese).

Shen, C.M., 2003. Millennial-scale variations and centennial-scale events in the southwest Asian Monsoon: Pollen evidence from Tibet (PhD Thesis). Louisianan State University, USA.

Shen, C.M., Liu, K.B., Tang, L.Y., Overpeck, J.T., 2006. Quantitative relationships between modem pollen rain and climate in the Tibetan Plateau. Review of Palaeobotany and Palynology 140, 61-77.

Shen, C.M., Tang, L.Y., Wang, S.M., 1996. Vegetation and climate during the past 22000 years in Zoige Region. Acta Micropalaeontologica Sinica 13, 401-406 (in Chinese with English abstract).

Shu, Q., Xiao, J.Y., Zhang, M.X., Zhao, Z.J., Chen, Y., Li, J.J., 2008. Climate change in northern Jiangsu Basin since the last interglacial. Geological Science and Technology Information 27, 59-64 (in Chinese with English abstract).

Stebich, M., Rehfeld, K., Schlütz, F., Tarasov, P.E., Liu, J., Mingram, J., 2015. Holocene vegetation and climate dynamics of NE China based on the pollen record from Sihailongwan Maar Lake. Quaternary Science Reviews 124, 275–289.

Sun, A.Z., Feng, Z.D., 2013. Holocene climatic reconstructions from the fossil pollen record at Qigai Nuur in the southern Mongolian Plateau. The Holocene 23, 1391-1402.

Sun, L.M., Xu, Q.H., Yang, X.L., Liang, W.D., Kong, Z.C., 2001. Vegetation and environmental changes in the Xuanhua Basin of Hebei Province since postglacial. Journal of Geomechanics 7, 303-308 (in Chinese with English abstract).

Sun, X.J., Du, N.Q., Chen, Y.S., Gu, Z.Y., Liu, J.Q., Yuan, B.Y., 1993. Holocene Palynological Recordsin Lake Selincuo, Northern Xizang. Acta Botanica Sinica 35, 943-950 (in Chinese with English abstract).

Sun, X.J., Du, N.Q., Weng, C.Y., Lin, R.F., Wei, K.Q., 1994. Paleovegetation and paleoenvironment of Manasi Lake, Xinjiang, N.W. China during the last 14 000 years. Quaternary Sciences 14, 239-248 (in Chinese with English abstract).

Tang, L.Y., Shen, C.M., 1996. Holocene pollen records of The Qinghai-Xizang Plateau. Acta Micropalaeontologica Sinica 13, 407-422 (in Chinese with English abstract).

Tang, L.Y., Shen, C.M., Liu, K.B., Overpeck, J.T., 1999. New high-resolution pollen records from two lakes in Xizang (Tibet). Acta Botanica Sinica 41, 896-902 (in Chinese with English abstract).

Tao, S.C., An, C.B., Chen, F.H., Tang, L.Y., Lv, Y.B., Zheng, T.M., 2009. Holocene vegetation changes interpreted from pollen records in Balikun Lake, Xinjiang, China. Acta Palaeontologica Sinica 48, 194-199 (in Chinese with English abstract).

Tarasov, P.E., Bezrukova, E.V., Krivonogov, S.K., 2009. Late glacial and Holocene changes in vegetation cover and climate in southern Siberia derived from a 15kyr long pollen record from Lake Kotokel. Climate of the Past Discussions 5, 127-151.

Telford, R.J., Birks, H.J.B., 2011. A novel method for assessing the statistical significance of quantitative reconstructions inferred from biotic assemblages. Quaternary Science Reviews 30, 1272-1278.

Volkov, I.A., Arkhipov, S.A., 1978. Quaternary deposits of the Novosibirsk Region. Joint Institute for Geology, Geophysics and Mineralogy, Siberia Branch, USSR Academy of Sciences, Novosibirsk (in Russian).

Wang, H.Y., Liu, H.Y., Cui, H.T., Abrahamsen, N., 2001. Terminal Pleistocene/Holocene palaeoenvironmental changes revealed by mineral-magnetism measurements of lake sediments for Dali Nor area, southeastern Inner Mongolia Plateau, China. Palaeogeography Palaeoclimatology Palaeoecology 170, 115-132.

Wang, M.H., 1987. The spore-pollen groups of peatland on Ruoergai Plateau and palaeobotany and paleoclimate. Scientia Geographica Sinica 7, 147-155 (in Chinese with English abstract).

Wang, W., Feng, Z., Ran, M., Zhang, C., 2013. Holocene climate and vegetation changes inferred from pollen records of Lake Aibi, northern Xinjiang, China: A potential contribution to understanding of Holocene climate pattern in East-central Asia. Quaternary International 311, 54-62.

Wang, Y., Herzschuh, U., Shumilovskikh, L.S., Mischke, S., Birks, H.J.B., Wischnewski, J., Böhner, J., Schlütz, F., Lehmkuhl, F., Diekmann, B., Wünnemann, B., Zhang, C., 2014. Quantitative reconstruction of precipitation changes on the NE Tibetan Plateau since the Last Glacial Maximum – extending the concept of pollen source area to pollen-based climate reconstructions from large lakes. Climate of the Past 10, 21–39.

Wang, Y., Wang, S.B., Jiang, F.C., Tong, G.B., 2003. Palynological Records in Xipu Section, Yangyuan. Journal of Geomechanics 9, 171-175 (in Chinese with English abstract).

Wang, Y., Wang, S.B., Zhao, Z.Z., Qin, Y., Ma, Y.S., Sun, J.M., Sun, H.Y., Tian, M.Z., 2005. Vegetation and environmental changes in Hexiqten Qi of Inner Mongolia in the past 16 000 years. Acta Geoscientica Sinica 26, 449-453 (in Chinese with English abstract).

Wen, Q.Z., Qiao, Y.L., 1990. Preliminary probe of climatic sequence in the last 13 000 years in Xinjiang Region. Quaternary Sciences 10, 363-371 (in Chinese with English abstract).

Wen, R.L., Xiao, J.L., Chang, Z.G., Zhai, D.Y., Xu, Q.H., Li, Y.C., Itoh, S., 2010. Holocene precipitation and temperature variations in the East Asian monsoonal margin from pollen data from Hulun Lake in northeastern Inner Mongolia, China. Boreas 39, 262-272.

Wischnewski, J., Mischke, S., Wang, Y., Herzschuh, U., 2011. Reconstructing climate variability on the northeastern Tibetan Plateau since the last Lateglacial – a multi-proxy, dual-site approach comparing terrestrial and aquatic signals. Quaternary Science Reviews 30, 82-97.

Xia, D.S., Ma, Y.Z., Chen, F.H., Wang, J.M., 1998. High-resolution vegetation and climate variations in Longxi Loess Plateau during Holocene. Journal of Lanzhou University (Natural Sciences) 34, 119-127 (in Chinese with English abstract).

Xiao, J.L., Xu, Q.H., Nakamura, T., Yang, X.L., Liang, W.D., Inouchif, Y., 2004. Holocene vegetation variation in the Daihai Lake region of north-central China: a direct indication of the Asian monsoon climatic history. Quaternary Science Reviews 23, 1669-1679.

Xiao, X.Y., Haberle, S.G., Shen, J., Yang, X.D., Han, Y., Zhang, E.L., Wang, S.M., 2014. Latest Pleistocene and Holocene vegetation and climate history inferred from an alpine lacustrine record, northwestern Yunnan Province, southwestern China. Quaternary Science Reviews 86, 35-48.

Xu, Q.H., Wu, C., Wang, Z.H., Tong, G.B., Wu, S.J., Zhang, J.P., Du, N.Q., Kong, Z.C., 1993. Approach to palaeo-environment in the west coast of Bohai Bay since 25000 a.B.P. Acta Phytoecologica et Geobotanica Sinica 17, 20-32 (in Chinese with English abstract).

Xu, Y.Q., 1998. The assemblage of Holocene spore pollen and its environment in Bosten Lake area, Xinjiang. Arid Land Geography 21, 43-49 (in Chinese with English abstract).

Yan, F.H., Ye, Y.Y., Mai, X.S., 1983. The sporo-pollen assemblage in the Luo 4 drilling of Lop Lake in Uygur Autonomous Region of Xinjiang and its significance. Seismology and Geology 5, 75-80 (in Chinese with English abstract).

Yan, S., Xu, Y.Q., 1992. The Holocene vegetation and environment in Daluoba Basin of Altay Mountains, Xinjiang. Xinjiang Geology 10, 279-284 (in Chinese with English abstract).

Yang, X.D., Wang, S.M., Tong, G.B., 1996. Character of palynology and changes of monsoon climate over the last 10000 years in Gucheng Lake, Jiangsu Province. Acta Botanica Sinica 38, 576-581 (in Chinese with English abstract).

Yang, X.L., Xu, Q.H., Zhao, H.P., 1999. Vegetation succession since last glacial in Taihang Mountains Area. Geography and Territorial Research 15, 81-88 (in Chinese with English abstract).

Yi, S.H., Saito, Y., Zhao, Q.H., Wang, P.X., 2003. Vegetation and climate changes in the Changjiang (Yangtze River) Delta, China, during the past 13,000 years inferred from pollen records. Quaternary Science Reviews 22, 1501-1519.

Yue, Y., Zheng, Z., Huang, K., Chevalier, M., Chase, B.M., Carré, M., Ledru, M.-P., Cheddadi, R., 2012. A continuous record of vegetation and climate change over the past 50,000 years in the Fujian Province of eastern subtropical China. Palaeogeography, Palaeoclimatology, Palaeoecology 365–366, 115-123.

Zhang, J.H., Kong, Z.C., Du, N.Q., 1997. An analysis of different sedimentary environment influence on pollen deposit in Beijing. Acta Sedimentologica Sinica 15, 57-63 (in Chinese with English abstract).

Zhang, Z.Y., Zhou, K.S., Yang, R.X., Zhang, S.L., Cai, Q.F., Lu, P., Hao, L.M., Wang, C., 2007. Environmental archaeology in the Shuangji River basin. Quaternary Research 27, 453-460 (in Chinese with English abstract).

Zhao, Y., Yu, Z., Zhao, W., 2011. Holocene vegetation and climate histories in the eastern Tibetan Plateau: controls by insolation-driven temperature or monsoon-derived precipitation changes? Quaternary Science Reviews 30, 1173-1184.

Zhao, Y., Yu, Z.C., Chen, F.H., Ito, E., Zhao, C., 2007. Holocene vegetation and climate history at Hurleg Lake in the Qaidam Basin, northwest China. Review of Palaeobotany and Palynology 145, 275-288.

Zhao, Z.M., Liu, A.M., Peng, W.， Ji, W.H., Li, R.S., Wang, Y.Z., Zhu, Y.T., 2007. Holocene environmental changes of northern Qinghai-Tibetan Plateau based on spore-pollen analysis. Arid Land Geography 30, 381-391 (in Chinese with English abstract).

Zhou, J., Liu, D.Y., Zhuang, Z.Y., Wang, Z.Z., Liu, L.D., 2008. The Sediment layers and the records of the paleoenvironment in the Chaoyanggang Lagoon, Rongcheng City of Shandong Province since Holocene transgression. Periodical of Ocean University of China 38, 803-808 (in Chinese with English abstract).

Zhou, J., Wang, S.M., Lv, J., 2003. Climatic and environmental changes from the sediment record of Erhai Lake over the past 10000 Years. Journal of Lake Sciences 15, 104-111 (in Chinese with English abstract).

Zhou, W.J., Yu, X.F., Jull, A.J.T., Burr, G., Xiao, J.Y., Lu, X.F., Xian, F., 2004. High-resolution evidence from southern China of an early Holocene optimum and a mid-Holocene dry event during the past 18,000 years. Quaternary Research 62, 39-48.

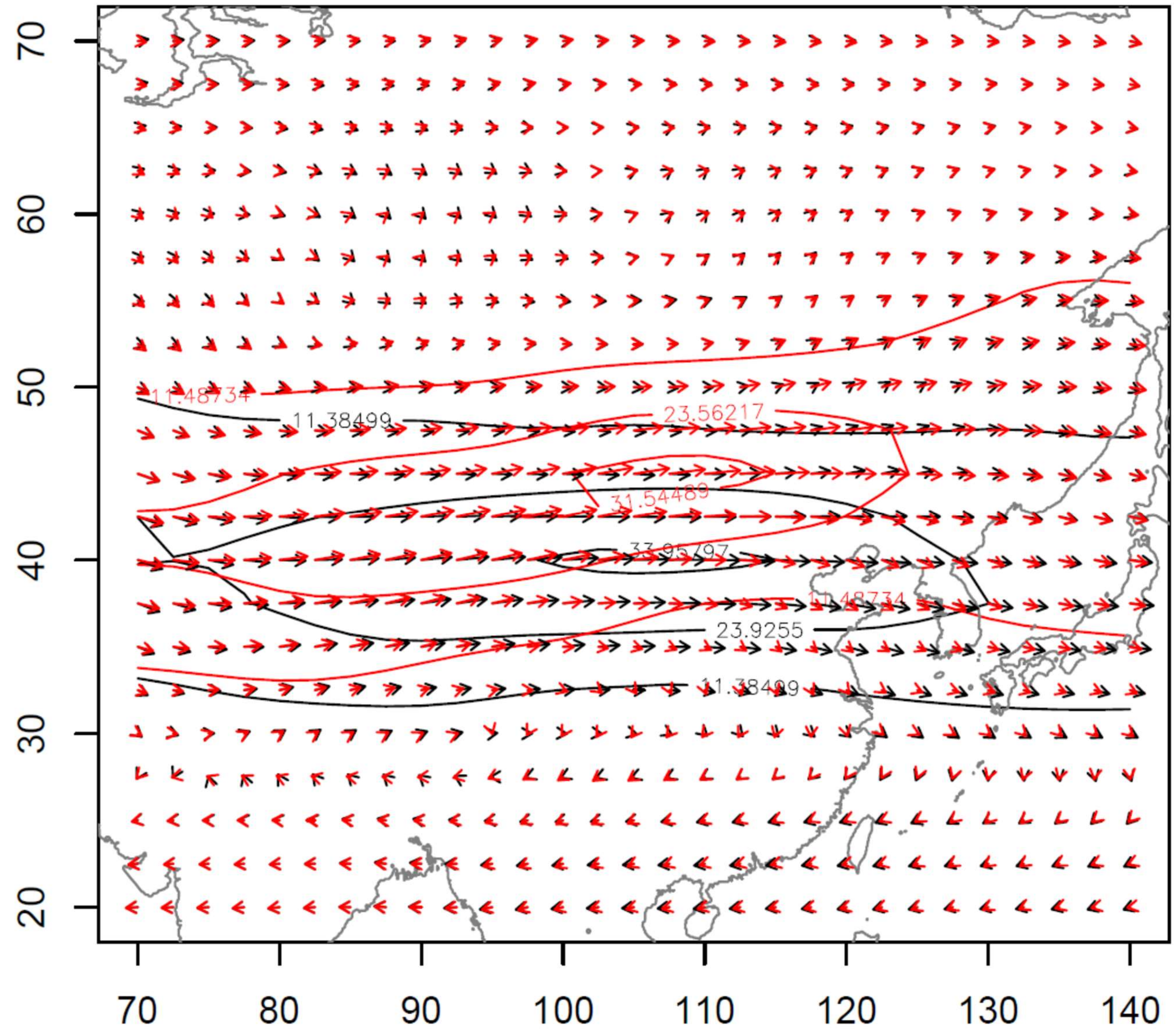

Supplementary Fig. 1: Modeled 250 hPa wind (and wind vectors) for 2 ka (black) and 9 ka (red) on the day of the year when the westerly wind stream reached its most northerly position, indicating the position and orientation of the westerly jet stream during the early Holocene compared with the late Holocene.

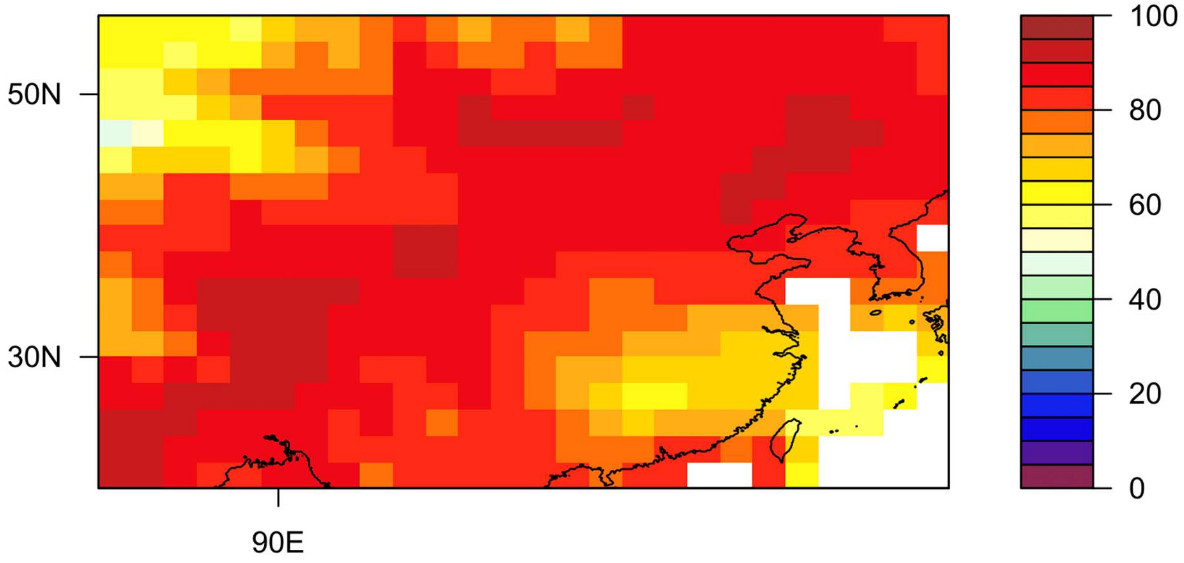

Supplementary Fig. 2: Percentage of precipitation during summer months (March to September) as calculated using the APHRODITE dataset (Yatagai et al., 2012). Results indicate that over the whole domain, 80% falls in the summer half year and that the summer contribution at all proxy sites (even in the northwest of the study area) is larger than 60%. (Even with respect to our modeling domain there is only one grid-cell in the northwestern-most part where winter precipitation dominates slightly. Southeastern China has a high share of autumn precipitation originating from to the southward transition of the westerly jet after summer.) Accordingly, we focus our argumentation on circulation mechanisms and related precipitation patterns during the summer (MAMJJAS).

Yatagai, A., Kamiguchi, K., Arakawa, O., Hamada, A., Yasutomi, N., Kitoh, A., 2012. APHRODITE: Constructing a Long-Term Daily Gridded Precipitation Dataset for Asia Based on a Dense Network of Rain Gauges. Bulletin of the American Meteorological Society 93, 1401-1415.

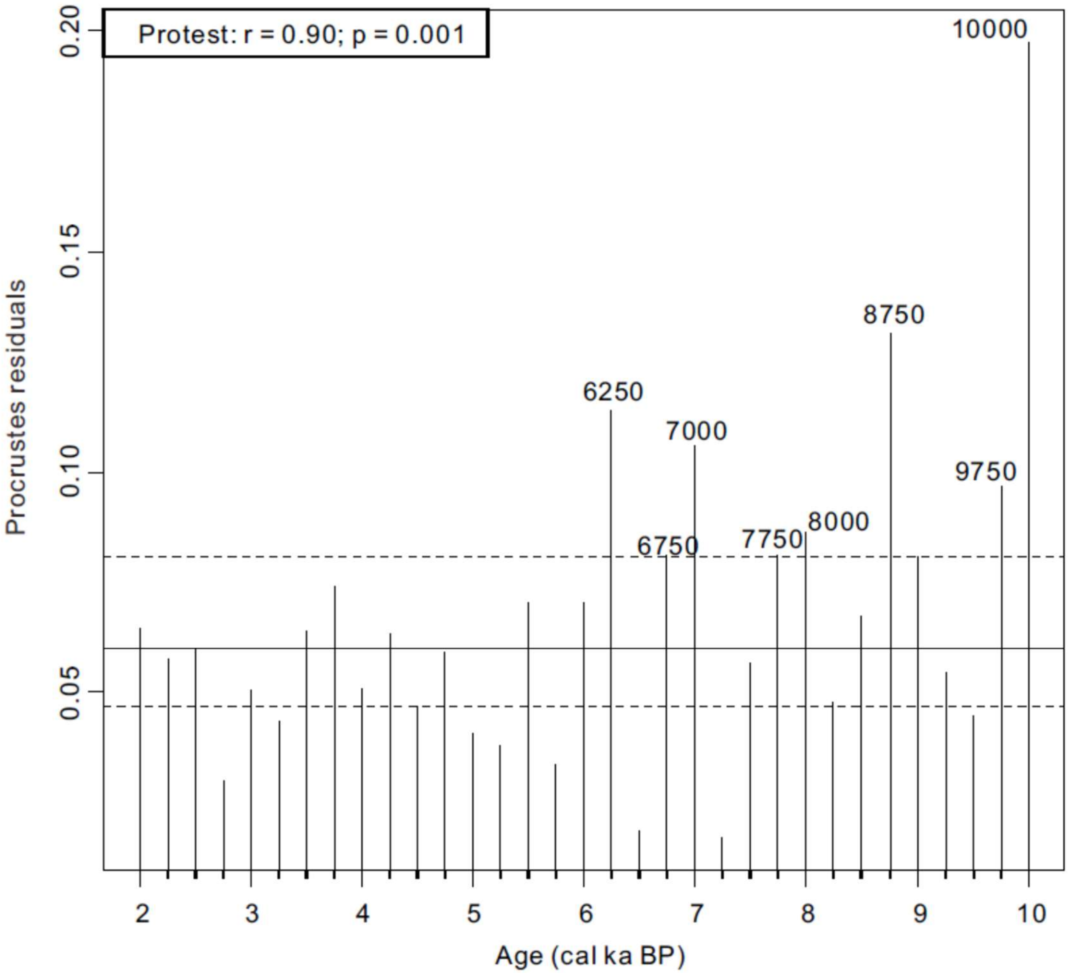

Supplementary Fig. 3: Comparison of pollen-based and non-pollen-based reconstructions. Non-pollen-based climate reconstructions were also available for 82 of the sites in the fossil pollen dataset. We extracted semi-quantitative moisture signals according to Herzschuh (2006) and Wang et al. (2010) from non-pollen proxies, including biological data (e.g. diatoms, ostracods, chironomidae), geochemical data (e.g. organic and inorganic carbon content, stable carbon and oxygen isotope ratios, elemental compositions), and geophysical data (e.g. grain size, magnetic susceptibility; Table 1). We translated the moisture signals at 500-year time intervals for 10–2 ka from these non-pollen studies into moisture signals on a five-part scale (−2, −1, 0, +1, +2), with the lowest value (−2) indicating the driest period for each site and the highest value (+2) indicating the wettest interval; the intermediate value (0) indicated moisture conditions similar to today. The moisture information is therefore on a relative scale and only tracks the changes at each individual site.

Multivariate datasets can be compared using Procrustes rotation, which assesses the overall degree of correlation between two or more ordination results and finds an optimal superimposition that maximizes their fit (Peres-Neto & Jackson, 2001). PROTEST performs a random permutation test and assesses the degree of concordance between two matrices, presenting the significance of the

Procrustes fit as an r-value with an associated p-value to indicate the likelihood of the relationship occurring by chance (Jackson, 1995). In our study we evaluated the similarity/dissimilarity between pollen-based and non-pollen-based inferences of moisture levels, using Procrustes rotation, and then tested the significance of any of the relationships detected using the associated PROTEST permutation test (Peres-Neto & Jackson, 2001) for non-metric multidimensional scaling (NMDS) results, covering the period from 10 to 2 cal. ka BP. The results are shown as residuals, with low residual values indicating a good agreement between datasets and high values indicating a weak agreement. The NMDS, Procrustes, and PROTEST (Peres-Neto & Jackson, 2001) analyses were carried out using the vegan package (Oksanen et al., 2012) in R 3.0.2 software (R Core Team, 2016).

References Supplementary Fig. 3

Herzschuh, U., 2006. Palaeo-moisture evolution at the margins of the Asian monsoon during the last 50 ka. Quaternary Science Reviews 25, 163–178.

Jackson, D.A., 1995. PROTEST: A PROcrustean Rendomization TEST of community environment concordance. Ecoscience 2, 297-303.

Oksanen, J., Blanchet, F.G., Kindt, R., Legendre, P., Minchin, P.R., O'Hara, R.B., Simpson, G.L., Solymos, P., Stevens, M.H.H., Wagner, H., 2012. vegan: Community Ecology Package, version 2.0-4. Available at: http://cran.r-project.org/web/packages/vegan//index.html.

Peres-Neto, P.R., Jackson, D.A., 2001. How well do multivariate datasets match? The advantages of a Procrustean superimposition approach over the Mantel test. Oecologia 129, 169-178.

R Core Team, 2016. R: A language and environment for statistical computing. R Foundation for Statistic Computing, Vienna.

Wang, Y., Liu, X., Herzschuh, U., 2010. Asynchronous evolution of the Indian and East Asian Summer Monsoon indicated by Holocene moisture pattern in monsoonal central Asia. Earth-Science Reviews 103, 135-153.

Supplementary Fig. 4 Correlation of the predicted and observed local climatological daily precipitation. Hashed areas are statistically significant (p=0.01)."

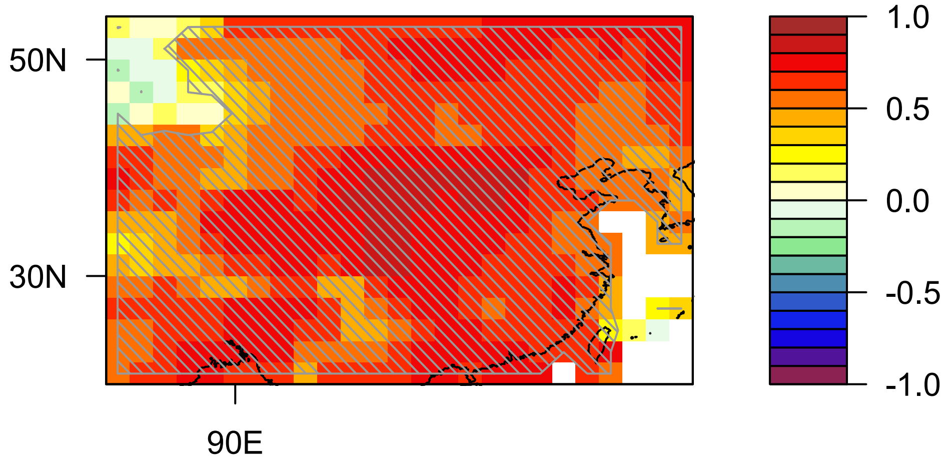

Supplement: Supplementary file 1 — Supplementary Information [file 41467_2019_9866_MOESM1_ESM.pdf]
