# Supplementary material for: Position and orientation of the westerly jet determined Holocene rainfall patterns in China
Source: Nat Commun. 2019 May 30;10:2376. doi: 10.1038/s41467-019-09866-8 (PMC6542844; doi:10.1038/s41467-019-09866-8)
Supplement: Supplementary file 2 — Supplementary Software [file 41467_2019_9866_MOESM2_ESM.pdf]

Supplementary Note 1: R code

Herzschuh et al Position and orientation of the westerly jet determined Holocene rainfall patterns in China

```
######R-code for Pann reconstruction
######useful package
library(rioja)  ###rioja version is 0.7-3
library(fields)
library(palaeoSig)

######data reading
######modern data
dat <- read.csv("mp+mc.csv", row.name=1)

######fossil data
all.fossil <- read.csv("fossil-pollen-data-101sites.csv")
id <- unique(all.fossil$ID)

######result files
recon.res <- all.fossil[, 1:8]
recon.res <- cbind(recon.res, "Pann", "Pann.err")
colnames(recon.res)[c(9, 10)] <- c("Pann", "Pann.err")
recon.res[, c(9, 10)] <- NA

note <- all.fossil[, 1:7]
note <- unique(note)
note <- cbind(note, NA, NA, NA, NA, NA, NA, NA)
colnames(note)[8:14] <- c("Sample", "Pann.min", "Pann.max", "Comp", "r2", "RMSEP", "Sig")

######main loop
```

```
distance <- 1000    #"distance" here means the extent of modern subset around the fossil site used in each reconstruction; km

for(i in id){fossil <- all.fossil[all.fossil$ID == i,]
        fossil.spp <- fossil[, 9:234]
        fossil.spp <- fossil.spp[, colSums(fossil.spp>0)>0]
        center <- cbind(fossil$Long[1], fossil$Lat[1])
        rdist <- rdist.earth(center, dat[,c("Long","Lat")], miles = FALSE)
        dat$rdist <- rdist[1,]
        dat1 <- dat[dat$rdist<distance,]
        note[note$ID==i,"Sample"] <- nrow(dat1)



        spp1 <- dat1[,1:161]
        spp1 <- spp1[,colSums(spp1>0)>0]
        pann <- dat1$Pann
        note[note$ID==i,"Pann.min"] <- min(pann)
        note[note$ID==i,"Pann.max"] <- max(pann)

        wapls <- crossval(WAPLS(sqrt(spp1), pann))
        t.t <- (rand.t.test(wapls))
        m <- 1
        note[note$ID==i,"Comp"] <- m
        note[note$ID==i,"r2"] <- t.t[m,2]
        note[note$ID==i,"RMSEP"] <- t.t[m,1]

        if(t.t[2,5] <= -5 & t.t[2,6] <= 0.05) m <- 2    #selection of the component
        pred<- predict(wapls, sqrt(fossil.spp), npls=m, sse = TRUE)
        recon.res[recon.res$ID==i,"Pann"] <- pred$fit[,m]
        recon.res[recon.res$ID==i,"Pann.err"] <- pred$SEP.boot[,m]

        print(i)
        }
```

```
write.csv(recon.res, file = "reconstruction.csv",row.names=FALSE)


######calculate the p value for samples between 2000 and 10000 a BP
#####modern data##########
dat <- read.csv("mp+mc.csv", row.name=1)


#####fossil data#########
all.fossil <- read.csv("fossil-pollen-data-101sites.csv")
all.fossil <- all.fossil[all.fossil$Cal.yr.BP >= 2000 & all.fossil$Cal.yr.BP <=10000,]
id <- unique(all.fossil$ID)


distance <- 1000    #"distance" here means the extent of modern subset around the fossil site used
in each reconstruction


for(i in id){fossil <- all.fossil[all.fossil$ID == i,]
        fossil.spp <- fossil[, 9:234]
        fossil.spp <- fossil.spp[, colSums(fossil.spp>0)>0]
        center <- cbind(fossil$Long[1], fossil$Lat[1])
        rdist <- rdist.earth(center, dat[,c("Long","Lat")], miles = FALSE)
        dat$rdist <- rdist[1,]
        dat1 <- dat[dat$rdist<distance,]


        spp1 <- dat1[,1:161]
        spp1 <- spp1[,colSums(spp1>0)>0]
        pann <- dat1$Pann
        wapls <- crossval(WAPLS(sqrt(spp1), pann))
        t.t <- (rand.t.test(wapls))
        m <- 1
        if(t.t[2,5] <= -5 & t.t[2,6] <= 0.05) m <- 2 #selection of the component
        pann.sig <- randomTF(spp = sqrt(spp1), env = data.frame(Pann = pann), fos =
sqrt(fossil.spp), n = 999, fun = WAPLS, col = m)
        note[note$ID==i,"Sig"] <- data.frame(pann.sig$sig)[1,1]
```

```
            print(i)
            }

write.csv(note, file = "model.statistic.csv")



######R-code for spatial interpolation
######dataset preparation
library(fields)
dat <- read.csv("reconstruction.csv")
id <- unique(dat$ID)

pann <- matrix(NA, 771, length(id))   #771 is the sample number from Gonghai Lake, the maximum
in the dataset
age <- matrix(NA, 771, length(id))
colnames(pann) <- id
colnames(age) <- id

for(i in id){dat1 <- dat[dat$ID == i, ]
            pann.m <- mean(dat1$Pann[dat1$Cal.yr.BP>=2000&dat1$Cal.yr.BP<=10000])
            for(j in 1:nrow(dat1)){age[j,as.character(i)] <- dat1$Cal.yr.BP[j]
                              pann.m <-
mean(dat1$Pann[dat1$Cal.yr.BP>=2000&dat1$Cal.yr.BP<=10000])
                              pann[j,as.character(i)] <- dat1$Pann[j]-pann.m
                              }
            }

write.csv(pann, file="pann.csv")
write.csv(age, file="age.csv")

######prepare the distance set
site <- read.csv("site101.csv")
```

```
rdist <- rdist.earth(site[,c("Long","Lat")], miles = FALSE)
rownames(rdist) <- id
colnames(rdist) <- id
write.csv(rdist, file="rdist.csv")

######function for spatial interpolation
K1D<-function(x,sigma) return(1/sqrt(2*pi)*exp(-x^2/(2*sigma^2)))
K2D<-function(x,y,sigma.x,sigma.y) return(K1D(x,sigma.x)*K1D(y,sigma.y))

######Read and remove the index row/column
m.age<-as.matrix(read.csv("age.csv",header=FALSE))
m.age<-m.age[-1,]
m.age<-m.age[,-1]

m.pann<-as.matrix(read.csv("pann.csv",header=FALSE))
m.pann<-m.pann[-1,]
m.pann<-m.pann[,-1]

rdist<-as.matrix(read.csv("rdist.csv",header=FALSE))
rdist<-rdist[-1,]
rdist<-rdist[,-1]

#Bandwith of smoother
kBandwidth.space=200  #Bandwith in space (km)
kBandwidth.time=1000  #Bandwith in time (yr)

#define the new time output
time.new<-seq(from=2000,to=10000,by=250)

nSites<-length(id)

result<-matrix(NA,nSites,length(time.new))
```

```
for (iSite in 1:nSites){print(paste("Working on site",iSite))
                    v.distance.space<-rdist[iSite,]  #Build the distance matrix by repeating the distance from site iSite to all other sites
                    m.distance.space<-rep(v.distance.space,dim(m.age)[1]) #convert it to a matrix
                    dim(m.distance.space)<-rev(dim(m.age))
                    m.distance.space<-t(m.distance.space)
                    for (iTime in 1:length(time.new)){m.distance.time<-abs(time.new[iTime]-m.age[,]) #Build the time matrix by substracting the output time from the proxy time
                                        weights1<-K2D(c(m.distance.space),c(m.distance.time),kBandwidth.space,kBandwidth.time) #Get the weights
                                        index<-!is.na(weights1)
                                        data.weighted<-c(m.pann)[index]*c(weights1)[index] #weight the data
                                        result[iSite,iTime]=sum(data.weighted)/sum(weights1[index])
                                        }
                    }

rownames(result) <- id
colnames(result) <- time.new
write.csv(result, file="spatial-interpolation.csv")

######R-code for Figure 1, pollen-based Pann part
######Fuzzy c-means clustering
library(e1071)
cm_data <-read.csv("spatial-interpolation.csv", row.name=1)
location <- read.csv("site101.csv")

######z transform
for(i in 1:nrow(cm_data)){a <- as.numeric(cm_data[i,])
                          a <- as.numeric(scale(a))
                          cm_data[i,] <-a
                          }
```

```
fcm <- cmeans(cm_data, 3, iter.max = 1000, method="cmeans", rate.par = 0.5, m=1.4)

center <- fcm$centers
membership <- cbind(location,fcm$membership)
colnames(membership)[c(5,6,7)] <- c("group_1","group_2","group_3")

write.csv(center, file="centers_pollen.csv",row.names=FALSE)
write.csv(membership, file="membership_pollen.csv",row.names=FALSE)

######results plotting
par(mfcol=c(3,2))
plot(fcm$centers[1,],ylim=c(-2,2), ylab="z-score Pann", col="red")
plot(fcm$centers[2,],ylim=c(-2,2), ylab="z-score Pann", col="green")
plot(fcm$centers[3,],ylim=c(-2,2), ylab="z-score Pann", col="blue",xlab="Age (cal ka BP)")

library(maps)
map(xlim=c(80,130),ylim=c(20,55),resolution=0, col="gray90",fill=TRUE)
map.axes()
points(membership$Long, membership$Lat, col=ifelse(membership[,"group_1"] >= 0.5, "red",NA),
pch=16, cex=2)

map(xlim=c(80,130),ylim=c(20,55),resolution=0, col="gray90",fill=TRUE)
map.axes()
points(membership$Long, membership$Lat, col=ifelse(membership[,"group_2"] >= 0.5, "green",NA),
pch=16, cex=2)

map(xlim=c(80,130),ylim=c(20,55),resolution=0, col="gray90",fill=TRUE)
map.axes()
points(membership$Long, membership$Lat, col=ifelse(membership[,"group_3"] >= 0.5, "blue",NA),
pch=16, cex=2)

######R-code for Figure 1 model-based Pann part
```

```
load("SourceDataFile3.dat")
model.result <- data.frame(sim.precip)
rownames(model.result) <- c(time(sim.precip))

######Location
lat <- seq(from=21,to=55,by=2)
long <- seq(from=80,to=130,by=2)
lats <- rep(lat, time=length(long))
lats <- lats[order(lats)]
longs <- rep(long, times=length(lat))

site <- colnames(model.result)
site.loc <- matrix(NA, ncol=3,nrow=length(site))
site.loc[,1] <- site
colnames(site.loc) <- c("site","Lat","Long")
site.loc <- as.data.frame(site.loc)
site.loc[,"Lat"] <- lats
site.loc[,"Long"] <- longs
site.loc <- as.data.frame(site.loc)

model.result <- t(model.result)
model.result <- model.result[complete.cases(model.result),]
site.loc <- site.loc[site.loc$site %in% rownames(model.result),]

######z transform
for(i in 1:nrow(model.result)){a <- as.numeric(model.result[i,])
                                a <- as.numeric(scale(a))
                                model.result[i,] <-a
                                }

fcm <- cmeans(model.result,3,iter.max = 1000, method="cmeans", rate.par = 0.5, m=1.4)
```

```
center <- fcm$centers
membership <- cbind(site.loc,fcm$membership)
colnames(membership)[c(4,5,6)] <- c("group_1","group_2","group_3")

write.csv(center, file="centers_model.csv",row.names=FALSE)
write.csv(membership, file="membership_model.csv",row.names=FALSE)

######results plotting
par(mfcol=c(3,2))
plot(fcm$centers[1,],ylim=c(-2,2), ylab="z-score Pann", col="red")
plot(fcm$centers[2,],ylim=c(-2,2), ylab="z-score Pann", col="green")
plot(fcm$centers[3,],ylim=c(-2,2), ylab="z-score Pann", col="blue",xlab="Age (cal ka BP)")

library(maps)
map(xlim=c(80,130),ylim=c(20,55),resolution=0, col="gray90",fill=TRUE)
map.axes()
points(membership$Long, membership$Lat, col=ifelse(membership[,"group_1"] >= 0.5, "red",NA),
pch=16, cex=2)

map(xlim=c(80,130),ylim=c(20,55),resolution=0, col="gray90",fill=TRUE)
map.axes()
points(membership$Long, membership$Lat, col=ifelse(membership[,"group_2"] >= 0.5, "green",NA),
pch=16, cex=2)

map(xlim=c(80,130),ylim=c(20,55),resolution=0, col="gray90",fill=TRUE)
map.axes()
points(membership$Long, membership$Lat, col=ifelse(membership[,"group_3"] >= 0.5, "blue",NA),
pch=16, cex=2)



######R-code for Loess in Figure 3
library(rioja)  #version is 0.7-3
```

```
dat <- read.csv("reconstruction.csv")
dat <- dat[dat$Cal.yr.BP>= 2000& dat$Cal.yr.BP<= 10000,]
dat$loess <- NA
id <- unique(dat$ID)

for(i in id){dat1 <- dat[dat$ID==i,]
            mod <- loess(dat1[,"Pann"] ~ dat1[,"Cal.yr.BP"],span=0.5)
            dat[dat$ID==i,"loess"] <- fitted(mod)
             }

write.csv(dat,file="reconstructionwithloessbetween2and10ka.csv")

######R-code for south China CCA
library(vegan)
dat <- read.csv("mp+mc.csv",row.name=1)
dat <- dat[dat$Lat <=30,]
spp <- dat[,c(1:161)]
spp <- spp[,sapply(spp,max)>=3&colSums(spp>0)>=3]
env <- dat[,c(166:200)]

(dca <- decorana(sqrt(spp)) )  #DCA
(mod<-cca(sqrt(spp)~Pann+Mtco+Mtwa+Tann, data = env))
vif.cca(mod) #delete Tann because of the highest VIF value

(mod<-cca(sqrt(spp)~Pann+Mtco+Mtwa, data = env))
vif.cca(mod)
anova(mod, by = "margin")

(mod<-cca(sqrt(spp)~Pann, data = env))
(mod<-cca(sqrt(spp)~Mtco, data = env))
(mod<-cca(sqrt(spp)~Mtwa, data = env))
```

```
######R-code for summer Pann CCA
library(vegan)
dat <- read.csv("mp+mc.csv",row.name=1)
spp <- dat[,c(1:161)]
spp <- spp[,sapply(spp,max)>=3&colSums(spp>0)>=3]
env <- dat[,c(166:200)]

(dca <- decorana(sqrt(spp)) )#DCA
(mod<-cca(sqrt(spp)~Pjja, data = env))
vif.cca(mod) #delete Tann

(mod<-cca(sqrt(spp)~Pann+Mtco+Mtwa, data = env))
vif.cca(mod)
anova(mod, by = "margin")

(mod<-cca(sqrt(spp)~Pann, data = env))
(mod<-cca(sqrt(spp)~Mtco, data = env))
(mod<-cca(sqrt(spp)~Mtwa, data = env))



######Correlation and significance testing of model to reconstructions
#basedrive="/Users/tlaepple/data/"
#path<-paste(basedrive,"paleoLibrary/src/",sep="")
#source(paste(path,"header.R",sep=""))
#setwd(paste(basedrive,"harvard/mgca/",sep=""))
#sourceDir("./src/common/speclib")
#setwd(paste(basedrive,"",sep=""))

######Set to the path of the dataset
#setwd("/Users/tlaepple/data/herzschuh_18_natcomm/data1")

library(PaleoSpec)
```

```
#function for spatial interpolation
K1D<-function(x,sigma) return(1/sqrt(2*pi)*exp(-x^2/(2*sigma^2)))
K2D<-function(x,y,sigma.x,sigma.y) return(K1D(x,sigma.x)*K1D(y,sigma.y))


##' @title Gaussian space&time kernel smoothing
##' @param m.age matrix[timepoints,id] of time in years
##' @param m.pann matrix[timepoints,id] of annual precip
##' @param rdist matrix of distances between every pair
##' @param time.new vector: new time axes in yr
##' @param kBandwidth.space #Bandwith in space (km)
##' @param kBandwidth.time #Bandwith in time (yr)
##' @return
##' @author Thomas Laepple
KernelSmooth<-function(m.age,m.pann,rdist,time.new=seq(from=2000,to=10000,by=250),kBandwidth.space=200,kBandwidth.time=1000)
                        {nSites<-length(site$Lat)
                         result<-matrix(NA,nSites,length(time.new))
                         for (iSite in 1:nSites){
                                print(paste("Working on site",iSite))
                                v.distance.space<-rdist[iSite,]  #Build the distance matrix by repeating the distance from site iSite to all other sites
                                m.distance.space<-rep(v.distance.space,dim(m.age)[1]) #convert it to a matrix
                                dim(m.distance.space)<-rev(dim(m.age))
                                m.distance.space<-t(m.distance.space)
                                for (iTime in 1:length(time.new)){m.distance.time<-abs(time.new[iTime]-m.age[,]) #Build the time matrix by substracting the output time from the proxy time
                                        weights1<-K2D(c(m.distance.space),c(m.distance.time),kBandwidth.space,kBandwidth.time) #Get the weights
                                        index<-!is.na(weights1)
                                        data.weighted<-c(m.pann)[index]*c(weights1)[index] #weight the data
```

```
result[iSite,iTime]=sum(data.weighted)/sum(weights1[index])
                                                  }
                                }
                    return(result)
                    }


##' @title Simplify the records by keeping the time-sorted, binning the years in to decades and removing duplicates
##' @param m.age  matrix[timepoints,id] of time in years
##' @param m.pann matrix[timepoints,id] of annual precip
##' @return list with modified m.age and new p.ann (list(m.age=out.age,m.pann=out.pann)))
##' @author Thomas Laepple
ReSortTime <- function(m.age,m.pann)
                {out.age <- m.age
                 out.age[]<-NA
                 out.pann<-m.pann
                 out.pann[]<-NA

                for (iIndex in 1:dim(m.age)[2])
                                        {index <- !is.na(m.age[,iIndex])
                                         age <- round(m.age[index,iIndex]/10)*10
                                         temp <- sort(age,index.return=TRUE)
                                         m.age[index,iIndex]<-temp$x
                                         m.pann[index,iIndex]<-m.pann[index,iIndex][temp$ix]

                                         index <- !is.na(m.age[,iIndex])
                                         age <- m.age[index,iIndex]
                                         pann <- m.pann[index,iIndex]

                                         indexNoDupl <-(!duplicated(age))
```

```
                                        out.age[1:sum(indexNoDupl),iIndex]<-
age[indexNoDupl]
                                        out.pann[1:sum(indexNoDupl),iIndex]<-
pann[indexNoDupl]

                                         }
            return(list(m.age=out.age,m.pann=out.pann))

            }

##' @title Create a surrogate dataset on the original resolution
##' @param m.age  matrix[timepoints,id] of time in years
##' @param m.pann matrix[timepoints,id] of annual precip
##' @param beta beta coefficient used in the simulation of the decadal time-series
##' @return matrix[timepoints,id] of surrogate annual precip
##' @author Thomas Laepple
CreateSurrogateMatrix <- function(m.age,m.pann,beta=1)
                    {output <- m.pann
                     output[]<-NA
                     for (iIndex in 1:dim(m.age)[2])
                                         {index <- !is.na(m.age[,iIndex])
                                          age <- m.age[index,iIndex]
                                          surrogate <-
ts(SimPowerlaw(beta,(max(age)/10)+100),start=-500,deltat=10)
                                          output[index,iIndex]<-
SubsampleTimeseriesBlock(surrogate,m.age[index,iIndex])
                                          }

                      return(output)

                       }


##' @title Correlation using output from Gaussian Smoother
```

```
##' @param result
##' @param site
##' @param sim.precip
##' @return
##' @author Thomas Laepple
corSmoothed<-function(result,site,sim.precip)
                    {for (i in 1:length(site$Lat))
                                                  {resultts<-
pTs(result[i,],time.new,lat=site$Lat[i],lon=site$Long[i])
                                                   resultModel<-
selspace.3D(sim.precip,lat1=site$Lat[i],lon1=site$Long[i])
                                                   save[i]<- cor(resultModel,resultts)
                                                   }
                      return(save)
                      }


##' @title Correlation using the raw data
##' @param m.data
##' @param site
##' @param sim.precip
##' @return
##' @author Thomas Laepple
corRaw<-function(m.data,site,sim.precip)
              {for (i in 1:length(site$Lat))
                                            {resultts <-
MakeEquidistant(na.omit(m.age[,i]),na.omit(m.data[,i]),time.target=1000*time(sim.precip))
                                             resultModel<-
selspace.3D(sim.precip,lat1=site$Lat[i],lon1=site$Long[i])
                                             save[i]<- cor(resultModel,resultts,use="pairwise.complete")
                                             }
               return(save)
               }


nPoints <- apply(m.age,2,function(x) sum(!is.na(x)))
```

```
##' @title Mean, weighted by nPoints
##' @param save
##' @return
##' @author Thomas Laepple
cw<-function(save) sum(save*(nPoints))/sum((nPoints))

#new time-vector (same as model simulationm)
time.new=seq(from=2000,to=10000,by=250)

#lat/lon
site <- read.csv("site101.csv")

#Read age, precip a distance data and remove the index row/column
m.age<-as.matrix(read.csv("age.csv",header=FALSE))
m.age<-m.age[-1,]
m.age<-m.age[,-1]

m.pann<-as.matrix(read.csv("pann.csv",header=FALSE))
m.pann<-m.pann[-1,]
m.pann<-m.pann[,-1]

#distance matrix
rdist<-as.matrix(read.csv("rdist.csv",header=FALSE))
rdist<-rdist[-1,]
rdist<-rdist[,-1]



#Clean up the data
temp<-ReSortTime(m.age,m.pann)
m.age <- temp$m.age
```

```
m.pann <- temp$m.pann



#Load the model simulation
load("minimalModelOutput.dat",verbose=TRUE)

resultts <- list()
resultModel<-list()
save<-vector()



N.R = 1000
sur.cor.raw<-matrix(NA,N.R,length(site$Lat))
for (i.R in 1:N.R){print(paste("Working in N.R:",i.R))
                        sm.pann <- CreateSurrogateMatrix(m.age,m.pann,beta=1)
                        sur.cor.raw[i.R,]<-corRaw(sm.pann,site,sim.precip)
                        }

obs.cor.raw<-corRaw(m.pann,site,sim.precip) #correlation of reconstruction
mean(obs.cor.raw)
sum(rowMeans(sur.cor.raw,na.rm=TRUE)>mean(obs.cor.raw,na.rm=TRUE))/dim(sur.cor.raw)[1]
#Empirical p-value

inN.R = 1000
sur.cor<-matrix(NA,N.R,length(site$Lat))
for (i.R in 1:N.R)
                      {print(paste("Working in N.R:",i.R))
                       sm.pann <- CreateSurrogateMatrix(m.age,m.pann,beta=1)
                       temp<-KernelSmooth(m.age,sm.pann,rdist,time.new=time.new)
                       sur.cor[i.R,]<-corSmoothed(temp,site,sim.precip)
                       }

int.pann<-KernelSmooth(m.age,m.pann,rdist,time.new=time.new)
```

```
obs.cor<-corSmoothed(int.pann,site,sim.precip) #correlation of reconstruction

sur.cor.weighted<-apply(sur.cor,1,cw)

cw(obs.cor)
sum(rowMeans(sur.cor)>mean(obs.cor))/dim(sur.cor)[1]  #Empirical p-value
sum(sur.cor.weighted>cw(obs.cor))/dim(sur.cor)[1]  #Empirical p-value

#save(sur.cor,sur.cor.raw,file="MC_2018_11_26.dat")
```
